# The shape function method of nonlinear thermal stress of granite fracture tips in a high-temperature environment

**DOI:** 10.1038/s41598-023-44570-0

**Published:** 2024-02-01

**Authors:** Yang Wang, Wen-hua Chen

**Affiliations:** 1 School of Civil Engineering, Beijing Jiaotong University, Beijing, 100044 People’s Republic of China; 2 China Academy of Cultural Heritage, Beijing, 100029 People’s Republic of China

**Keywords:** Engineering, Civil engineering

## Abstract

Exposed rock masses in tunnel portals are susceptible to thermal deterioration in southern China, where temperatures are relatively high. The thermal stress field of rock masses is affected by fracture shape and distribution as fractures near the surface are channels for solar radiation energy to be converted into rock thermal energy. In this study, a function expression is developed for triangular heat sources of fractured rock masses in a tunnel portal in a high-temperature environment. By the function expression, the temperature field and thermal stress field are calculated, and the influence of fracture shape parameters and multi-fracture interaction is analyzed. The results are as follows: (1) the temperature field and thermal stress field of exposed rocks are redistributed by fractures. The internal temperature of the fractured rocks is higher than that of non-fractured rocks, and thermal stress near the fracture tip increases. (2) For triangular fractures of the same length, thermal stress increases as the apex angle increases. (3) When the spacing between parallel fractures or coplanar fractures is close, the superposition effect of thermal stress becomes significant. (4) In a high-temperature environment, temperature field and thermal stress field of a fractured rock are both nonlinear as temperature and thermal stress around fractures increase significantly. The results provide effective reference for stability evaluation of fractured rock masses in tunnel portals and offer theoretical foundation for thermal diseases analysis and protection measures of tunnel engineering in high-temperature environments of southern China.

## Introduction

**Figure 1:**
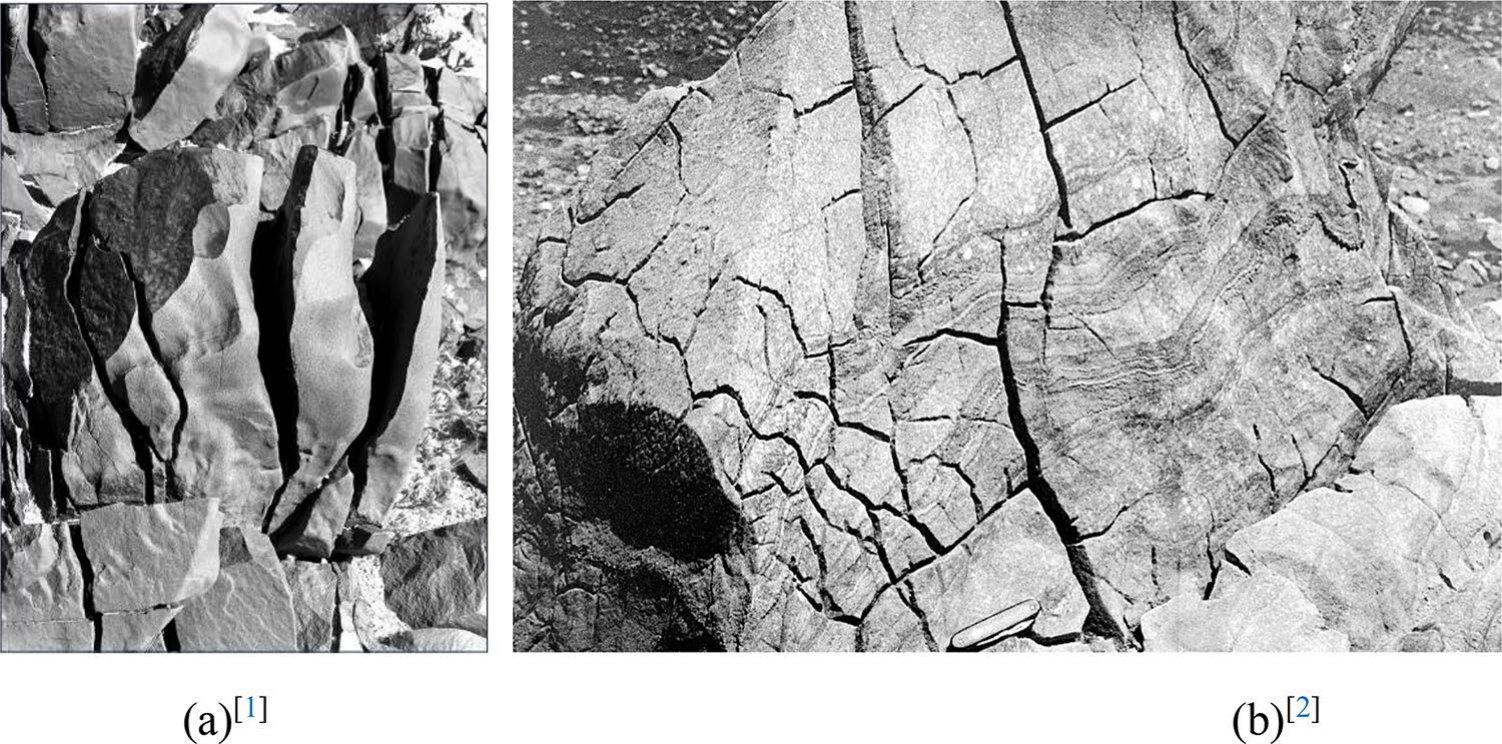
Thermal cracking of exposed rocks in high-temperature environments.

High-temperature environments play an important role in the thermal effect of exposed rock masses^1,2^ (Fig. 1). Rock masses in tunnel portals are susceptible to thermal deterioration in southern China, where high temperature lasts for a long time and solar radiation is strong. It is particularly important to note in the southeastern coastal areas that the average annual temperature is 22–25 °C and the average annual total solar radiation is 4400-5000 M J/m^2^. The rocks directly exposed to sunlight can have a surface temperature of 65 °C, or even higher, during hot weather. Thermal stress occurs and fractures develop and expand in exposed rocks due to the cumulative effect of temperature fluctuations, affecting the structural stability of tunnels, hampering the flow of traffic and causing enormous economic losses. Studying the thermal effect of fractured rock masses in a tunnel portal in high-temperature environments is of great significance as high temperature occurs even frequently due to global warming.

In high-temperature environments, the temperature field of the exposed rocks changes periodically under the influence of insolation^3–9^. Numerous types of research have been conducted on heat-induced cracking of rocks caused by insolation. As early as 1869, several scholars proposed that the temperature changes of rocks resulting from insolation were responsible for rock failure and cracking. Merill’s test data indicates thermal stress of rocks generated by insolation is approximately 0.35 to 6.33Mpa^10^, which is sufficient for rock cracking. Hall^2^ illustrated that heat exchange generated by insolation on the surface of a rock leads to thermal stress, resulting in rock cracking. Wang and Chen^11,12^ proposed a calculation method for thermal stress of fractured rocks under solar radiation based on nonlinearity of thermal conduction properties of fractured rock tips. The results show that fractures change the thermal stress field of rocks, and the thermal stress of a fractured rock tip increases. Wedekind et al.^13^ demonstrated that sandstone weathering is the main deterioration characteristic of the Angkor monuments in Cambodia, and studied the thermal expansion process of architectural sandstone. The results show that the tensile stress caused by solar radiation and thermal expansion is the main reason for cracking of sandstone of the Phnom Bakheng Temple. Collins and Stock^14^ believed that the diurnal cycle of solar radiation and environmental temperature leads to expanding of rock fractures. Ding et al.^15^ studied the influence of insolation and slope aspect on the development of debris flow. The difference of solar radiation intensity between shady slopes and sunny slopes is the reason for the difference of rock cracking. Kompanikova et al.^16^ measured the changes in physical properties and mineral composition of two types of sandstone exposed to sunlight and high temperature. The results show that mineral composition and porosity are the two main factors affecting the damage and destruction of natural stone under high temperature. Field measurements and simulation tests were conducted by Eppes et al.^17^ to study the insolation-induced fractures of rocks and fracture distribution characteristics.

The temperature field and thermal stress field of exposed rocks could be changed by fractures as solar radiation transfer into the interior of the rocks and converted into thermal energy through fractures. Thus, the shape and distribution of fractures have a great influence on the thermal stress field of rocks. The fracture planes are approximately triangular^18–21^ in shape and easily expandable because there are usually unstable blocks formed by the intersection of fracture planes and facing planes in shallow rocks. Accordingly, the triangle is the typical shape for studying the stability of the fractured rocks. Furthermore, rocks contain many fractures that are distributed randomly. Interaction of multiple fractures causes a superposition of stress fields of fracture tips^22–26^. An analysis of the thermal stress field based on a fracture shape of multi-fractured rocks is essential to evaluating rock stability in high-temperature environments.

Thermal deterioration of exposed rock masses in tunnel portals cannot be ignored in high-temperature areas of southern China. Due to a lack of research on thermal cracking mechanisms, although various measures have been taken to protect the rock masses from thermal deterioration in tunnel engineering, they are often ineffective. Insolation is a significant contributing factor to rock deterioration. The previous studies on the thermal effects of rocks have mostly concentrated on low-temperature fields around 0 °C^27–30^ and extremely high-temperature fields^31–41^ but rarely considered the influence of climate and environment. Shape and location of fractures affect the temperature field and thermal stress field of rocks in the high-temperature environments. However, no research has been conducted to investigate the thermal effect of fractured rocks based on fracture shape. In this study, a function expression of triangular heat sources is established for fractured rocks in high natural environment temperature using Green's function method and mirror image method. By the function expression, the temperature field and thermal stress field of fracture tips of a rock are calculated, and the influence of fracture shape parameters and fracture interaction is analyzed. The results provide an effective reference for evaluating the stability of fractured rock masses in a tunnel portal and offer a theoretical foundation for analyzing the thermal diseases and establishing protection measures of tunnel engineering in high temperature areas of southern China.

## The thermal stress field of fracture tip of a rock in a high-temperature environment

### The thermal effect of a fractured rock in a high-temperature environment

In a high-temperature environment, the process of heat exchange between exposed rocks and the external environment is considered a comprehensive effect of solar radiation, radiation heat transfer and convection heat transfer on the surface of a semi-infinite solid. When there are surface fractures in a rock, the heat exchange process presents that energy of solar radiation and atmospheric radiation is absorbed by fractures and converted into thermal energy of rock, and convection heat transfer is unavailable as the air in fractures does not flow.

**Figure 2:**
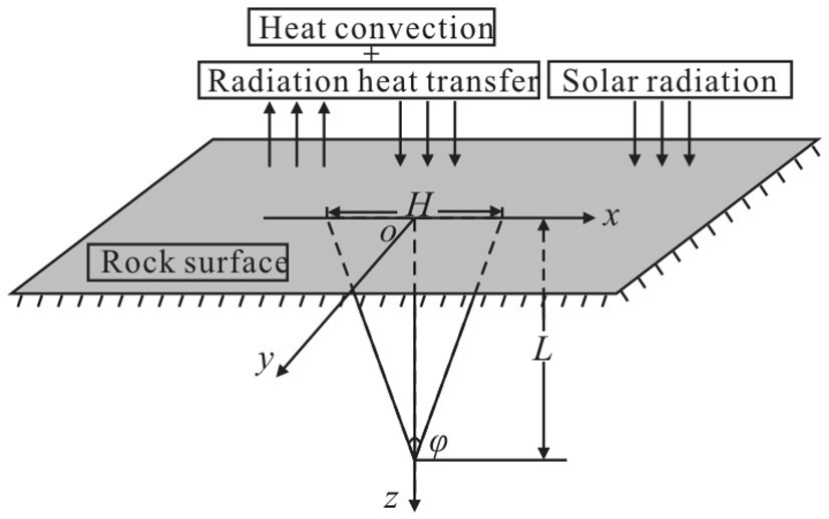
The heat exchange between a triangular fractured rock and the external environment^11,12^.

The heat exchange between a fractured rock and the external environment is shown in Fig. 2. Radiation energy is absorbed and reflected by fracture fronts and then decays. Absorption and reflection repeat several cycles in a fracture and then all radiation energy has been absorbed or emitted through the fracture, as fracture shapes are long and narrow, and fracture apertures are small. The solar radiation absorption coefficient is defined as the ratio of solar radiation energy absorbed by a rock to the total solar radiation energy reaching the rock. The absorption coefficient of rock to solar short-wave radiation and atmospheric long-wave radiation is 0.85 and 0.95, respectively^42^. After repeated absorption and reflection, the absorption coefficient is close to 1, meaning that fracture fronts absorb all heat. Due to the small width of a fracture compared to a semi-infinite rock, a fracture front can be considered as a finite line heat source with uniform energy density.

As a basis for establishing a heat source function expression of a fractured rock, the following assumptions can be made: (1) The energy density of solar radiation to rocks is uniform. (2) A rock is a homogeneous continuous medium. Its isotropic thermal conductivity properties and physical–mechanical properties do not change with temperature as the temperature variation of rocks in natural environment is very small. (3) Fractures are open and connected to the atmosphere without filling. (4) The fracture surface is triangle in shape and perpendicular to ground surface. (5) The heat absorbed by fracture fronts (two sides of a triangular fracture) is uniformly distributed during the process of heat transfer.

### The temperature field of fracture tip of a rock

The thermal effect of exposed rocks in high-temperature environments is temperature variation caused by heat flux transmitted through medium at the boundary of half-space. According to the theory of heat conduction, rock temperature can be expressed as

1 $$ \frac{\partial T}{{\partial t}} = a\left( {\frac{{\partial^{2} T}}{{\partial x^{2} }} + \frac{{\partial^{2} T}}{{\partial y^{2} }} + \frac{{\partial^{2} T}}{{\partial z^{2} }}} \right) $$

where *T* is temperature (°C), *t* is time (s), *a* = *λ*/*ρc* is thermal diffusivity of a rock (m^2^ s^−1^), *λ*, *ρ*, *c* are thermal conductivity (W m^−1^ K^1^), density (kg m^−3^) and heat capacity (J kg^−1^ K^−1^) of a rock, respectively.

As a result, the following formula can be obtained

2 $$ \frac{\partial \Delta T}{{\partial t}} = a\left( {\frac{{\partial^{2} \Delta T}}{{\partial x^{2} }} + \frac{{\partial^{2} \Delta T}}{{\partial y^{2} }} + \frac{{\partial^{2} \Delta T}}{{\partial z^{2} }}} \right) $$

where Δ*T* is temperature difference (°C), $\Delta T = T - T_{0}$, *T*_0_ is initial rock temperature (°C).

The boundary condition of heat exchange between a rock surface and an environment could be expressed as

3 $$ \left. { - \lambda \frac{\partial \Delta T}{{\partial z}}} \right|_{z = 0} = q_{1} $$

where $q_{1} = \alpha^{\prime } q_{s} + \varepsilon q_{r} + q_{h}$, *q*_*s*_, *q*_*r*_, *q*_*h*_ are heat flux density of solar radiation, radiation heat transfer and convection heat transfer (W m^−2^), respectively^42^. *α*′ and *ε* are absorption coefficients of a rock to solar radiation and atmospheric radiation.

Effective radiation $F = \varepsilon q_{r}$ is determined by utilizing the method of expanding temperature amplitude. Thus, air temperature function could be expressed as:

4 $$ T_{a}^{{\prime }} = T_{M} + \left( {T_{A} + C_{f} \alpha^{{\prime }} } \right)\sin \left[ {\frac{{\left( {t - t_{0} } \right)\uppi }}{12}} \right] $$

where *T*_*M*_ is mean daily temperature (°C), *T*_*A*_ is daily temperature amplitude (°C), *t*_0_ is time parameters of maximum and minimum temperature, *C*_*f*_ is effective radiation influence coefficient^43^.

Analytical solution of temperature field of unsteady heat conduction in a semi-infinite solid under the second boundary condition could be obtained by^42^:

5 $$ \Delta T_{1} = \frac{{2\alpha^{{\prime }} q_{s} \sqrt {{{at} \mathord{\left/ {\vphantom {{at}\uppi }} \right. \kern-0pt}\uppi }} }}{\lambda }\exp \left( { - \frac{{z^{2} }}{4at}} \right) - \frac{{\alpha^{{\prime }} q_{s} z}}{\lambda }{\text{erf}}\left( {\frac{z}{{2\sqrt {at} }}} \right) $$

Analytical solution under the third boundary condition could be obtained by:

6 $$ \Delta T_{2} = \left[ {{\text{erf}}\left( {\frac{z}{{2\sqrt {at} }}} \right) - \exp \left( {\frac{hz}{\lambda } + \frac{{h^{2} at}}{{\lambda^{2} }}} \right){\text{erfc}}\left( {\frac{z}{{2\sqrt {at} }} + \frac{{h\sqrt {at} }}{\lambda }} \right)} \right]\left( {T_{a}^{{\prime }} - T_{0} } \right) $$

where *h* is convective heat transfer coefficient of a rock (W m^−2^ K^−1^).

In a high-temperature environment, temperature field of a rock without fracture could be written as:

7 $$ \overline{T} = \Delta T_{1} + \Delta T_{2} + T_{0} $$

**Figure 3:**
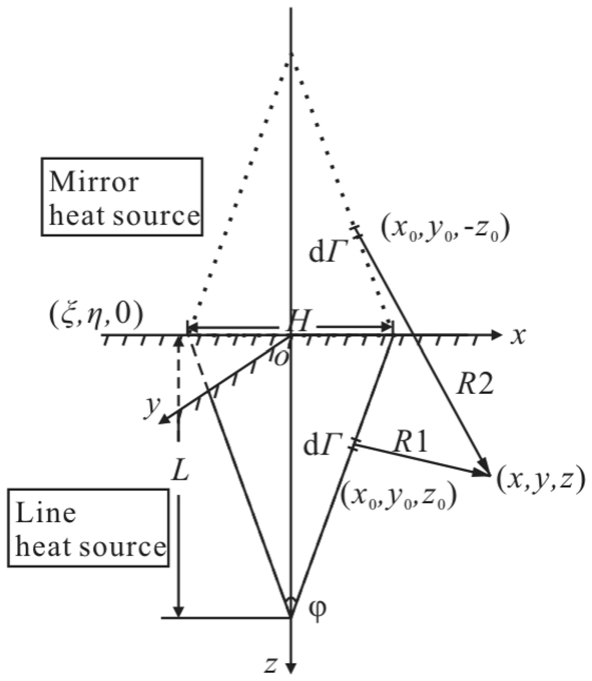
Heat source model.

When there are near surface fractures in a rock, the fracture fronts can be considered as finite line heat sources with uniform heat flux as fractures absorb radiation energy and convert it into heat energy (Fig. 3). The boundary condition is that the temperature of medium surface (the ground temperature) is uniform and equal to the temperature of medium at infinity (the initial rock temperature *T*_0_).

On a fracture front:

8 $$ \left. { - \lambda \frac{\partial \Delta T}{{\partial z}}} \right|_{\Gamma } = q_{2} $$

where $q_{2} = q_{s} + q_{a}$, *q*_*a*_ is heat flux density of atmospheric radiation (W m^−2^)^42^.

The Green’s Function Solution of temperature increment produced by a unit instantaneous point heat source (*x*_0_, *y*_0_, *z*_0_) at *t*_0_ in an infinite rock could be expressed as follow^44^:

9 $$ \Delta T^{0} = \frac{1}{{\rho c\left[ {4a\uppi \left( {t - t_{0} } \right)} \right]^{{{3 \mathord{\left/ {\vphantom {3 2}} \right. \kern-0pt} 2}}} }}\exp \left( { - \frac{{R^{2} }}{{4a\left( {t - t_{0} } \right)}}} \right) $$

where Δ*T*_0_ is temperature difference generated by a point heat source (°C), *R* is distance from the point heat source, $R = \sqrt {\left( {x - x_{0} } \right)^{2} + \left( {y - y_{0} } \right)^{2} + \left( {z - z_{0} } \right)^{2} }$.

Temperature increment produced by a point heat source (*x*_0_, *y*_0_, *z*_0_) with intensity *q*_2_ in time period $t_{m - 1} \sim t_{m}$ can be given by:

10 $$ \Delta T^{ * } = \frac{{q_{2} }}{{4\uppi \lambda R}}{\text{erfc}}\left( {\sqrt {\frac{{R^{2} }}{{4a\left( {t_{m} - t_{m - 1} } \right)}}} } \right) $$

The time of diurnal insolation is divided into *M* hours. If intensity of heat source remains constant in the *m*th hour with value of *q*_2_(*t*_*m*_), temperature increment of a fractured rock during the insolation period can be given by:

11 $$ \Delta T^{t} = \sum\limits_{m = 1}^{M} {q_{2} \left( {t_{m} } \right)\Delta T^{ * } } $$

Temperature increment caused by a triangular fracture line heat source can be given by:

12 $$ \Delta T^{tl} = \int_{\Gamma } {\Delta T^{t} } {\text{d}}\Gamma $$

where *Γ* is a triangular fracture line heat source, $\Gamma {:}z = - x\cot \left( {{\varphi \mathord{\left/ {\vphantom {\varphi 2}} \right. \kern-0pt} 2}} \right) + L\left( {0 < x \le {H \mathord{\left/ {\vphantom {H 2}} \right. \kern-0pt} 2}} \right),z = x\cot \left( {{\varphi \mathord{\left/ {\vphantom {\varphi 2}} \right. \kern-0pt} 2}} \right) + L\left( { - {H \mathord{\left/ {\vphantom {H 2}} \right. \kern-0pt} 2}} \right.\left. { \le x < 0} \right)$, *φ* is apex angle of a triangular fracture (°), *L* is length of a fracture (m), *H* is length of a fracture opening (m), the position relationship is shown in Fig. 3.

Using the mirror image method, a mirror heat source with an intensity of − *q*_2_(*t*_*m*_) is set at a position symmetrical to the line heat source $\Gamma {:}z = x\cot \left( {{\varphi \mathord{\left/ {\vphantom {\varphi 2}} \right. \kern-0pt} 2}} \right) - L\left( {0 < x \le {H \mathord{\left/ {\vphantom {H 2}} \right. \kern-0pt} 2}} \right),z = - x\cot \left( {{\varphi \mathord{\left/ {\vphantom {\varphi 2}} \right. \kern-0pt} 2}} \right) - L\left( {{{ - H} \mathord{\left/ {\vphantom {{ - H} 2}} \right. \kern-0pt} 2} \le x < 0} \right)$. Temperature increment caused by superposition of all *k* heat sources (line heat sources and mirror heat sources) can be given by:

13 $$ \Delta T_{3} = \sum\limits_{l = 1}^{k} {\Delta T^{tl} } $$

In a high-temperature environment, temperature field of fracture tip of a rock can be obtained by:

14 $$ \overline{\overline{T}} = \Delta T_{1} + \Delta T_{2} + \Delta T_{3} + T_{0} $$

### The thermal stress field of fracture tip of a rock

Thermal stress occurs in an exposed rock due to the temperature distribution is not uniform and the thermal expansion and contraction of each point of a rock are inconsistent under influence of insolation in high-temperature environments. Temperature change caused by rock deformation is ignored and coupling term of heat conduction equation is not taken into account because the temperature change is slow. Equilibrium equation of a rock is

15 $$ \sigma_{ij,i} = 0 $$

where *σ*_*ij*_ is thermal stress of a rock (MPa), *i* and *j* take *x*, *y* or *z*.

When temperature of a rock changes, the constitutive equation is

16 $$ \sigma_{ij} = \frac{E}{1 + \mu }\varepsilon_{ij} + \frac{{\delta_{ij} E}}{1 - 2\mu }\left[ {\frac{\mu }{{\left( {1 + \mu } \right)}}\varepsilon_{v} - \alpha \Delta T} \right] $$

where $\varepsilon_{ij} = {{\left( {u_{i,j} + u_{j,i} } \right)} \mathord{\left/ {\vphantom {{\left( {u_{i,j} + u_{j,i} } \right)} 2}} \right. \kern-0pt} 2}$ is strain of a rock, *u* is displacement of a rock (m), *ε*_*v*_ is volumetric strain, *E* is modulus of elasticity of a rock (MPa), *μ* is Poisson’s ratio, *α* is coefficient of thermal expansion of a rock (1/K), *δ*_*ij*_ is Kronecker delta function.

Boundary condition of a semi-infinite rock is

17 $$ \left\{ {\begin{array}{*{20}l} {\left. {\sigma_{ij} } \right|_{t = 0} = \left. {\sigma_{ij} } \right|_{z = 0} = 0} \hfill \\ {\left. {u_{i} } \right|_{t = 0} = \left. {u_{i} } \right|_{z \to \infty } = 0} \hfill \\ \end{array} } \right. $$

In a high-temperature environment, stress field of an exposed non-fractured rock resulting from thermal effect can be expressed as^45^:

18 $$ \overline{\sigma }_{ij} = \left\{ {\begin{array}{*{20}l} { - \delta_{ij} \frac{\alpha E}{{1 - \mu }}\Delta T} \hfill & {\quad \left( {i,j = x\;{\text{or}}\;y} \right)} \hfill \\ 0 \hfill & {\quad \left( {i\;{\text{or}}\;j = z} \right)} \hfill \\ \end{array} } \right. $$

When there are near surface fractures in a rock, fracture fronts are regarded as finite line heat sources with uniform heat flux. The Green's Function Solution of thermal stress field caused by a unit instantaneous point heat source (*x*_0_, *y*_0_, *z*_0_) at *t*_0_ in an infinite rock could be expressed as^45^:

19 $$\begin{aligned}   \sigma _{{ij}}^{0}  &  = \frac{{\alpha E}}{{4\pi \lambda R^{3} \left( {1 - \mu } \right)}}\left\{ {\delta _{{ij}} \left[ {{\text{erf}}\left( {R/\sqrt {4a\left( {t - t_{0} } \right)} } \right) - \frac{{R\,{\text{exp}}\left( { - R^{2} /4a\left( {t - t_{0} } \right)} \right)}}{{\sqrt {\pi a\left( {t - t_{0} } \right)} }} - \frac{{R^{3} \,\exp \left( { - R^{2} /4a\left( {t - t_{0} } \right)} \right)}}{{2\sqrt \pi  \left[ {a\left( {t - t_{0} } \right)} \right]^{{{3 \mathord{\left/ {\vphantom {3 2}} \right. \kern-\nulldelimiterspace} 2}}} }}} \right]} \right. \\    &\left. {  \quad  - \frac{{\left( {x_{i}  - x_{{i0}} } \right)\left( {x_{j}  - x_{{j0}} } \right)}}{{R^{2} }}\left[ {3{\text{erf}}\left( {R/\sqrt {4a\left[ {a\left( {t - t_{0} } \right)} \right]} } \right) - \frac{{3R\,{\text{exp}}\left( { - R^{2} /4a\left( {t - t_{0} } \right)} \right)}}{{\sqrt {\pi a\left( {t - t_{0} } \right)} }} - \frac{{R^{3} \,\exp \left( { - R^{2} /4a\left( {t - t_{0} } \right)} \right)}}{{2\sqrt \pi  \left[ {a\left( {t - t_{0} } \right)} \right]^{{{3 \mathord{\left/ {\vphantom {3 2}} \right. \kern-\nulldelimiterspace} 2}}} }}} \right]} \right\} \\  \end{aligned}$$

where $\sigma_{ij}^{0}$ is thermal stress generated by a point heat source (MPa), *i* and *j* take *x*, *y* or *z*.

Thermal stress field generated by a point heat source (*x*_0_, *y*_0_, *z*_0_) with intensity *q*_2_ in time period $t_{m - 1} \sim t_{m}$ can be given by:

20 $$ \begin{aligned} \sigma_{ij}^{ * } & = \frac{{\alpha Eq_{2} }}{{8\pi \lambda R\left( {1 - \mu } \right)}}\left\{ {\delta_{ij} \left[ {\frac{{2at{\text{erf}}\left( {{R \mathord{\left/ {\vphantom {R {\sqrt {4a\left( {t_{m} - t_{m - 1} } \right)} }}} \right. \kern-0pt} {\sqrt {4a\left( {t_{m} - t_{m - 1} } \right)} }}} \right)}}{{R^{2} }}} \right.} \right. - \frac{{\sqrt {4a\left( {t_{m} - t_{m - 1} } \right)} \exp \left( { - {{R^{2} } \mathord{\left/ {\vphantom {{R^{2} } {4a\left( {t_{m} - t_{m - 1} } \right)}}} \right. \kern-0pt} {4a\left( {t_{m} - t_{m - 1} } \right)}}} \right)}}{R\sqrt \pi } \\ & \quad \left. { - {\text{erfc}}\left( {\frac{R}{{\sqrt {4a\left( {t_{m} - t_{m - 1} } \right)} }}} \right)} \right] - \frac{{\left( {x_{i} - x_{i0} } \right)\left( {x_{j} - x_{j0} } \right)}}{{R^{2} }}\left[ {\frac{{6a\left( {t_{m} - t_{m - 1} } \right){\text{erf}}\left( {{R \mathord{\left/ {\vphantom {R {\sqrt {4a\left( {t_{m} - t_{m - 1} } \right)} }}} \right. \kern-0pt} {\sqrt {4a\left( {t_{m} - t_{m - 1} } \right)} }}} \right)}}{{R^{2} }}} \right. \\ & \quad - \frac{{3\sqrt {4a\left( {t_{m} - t_{m - 1} } \right)} \exp \left( { - {{R^{2} } \mathord{\left/ {\vphantom {{R^{2} } {4a\left( {t_{m} - t_{m - 1} } \right)}}} \right. \kern-0pt} {4a\left( {t_{m} - t_{m - 1} } \right)}}} \right)}}{R\sqrt \pi }\left. {\left. { + {\text{erfc}}\left( {\frac{R}{{\sqrt {4a\left( {t_{m} - t_{m - 1} } \right)} }}} \right)} \right]} \right\} \\ \end{aligned} $$

The time of diurnal insolation is divided into *M* hours. If heat source intensity remains constant in the *m*th hour with a value of *q*_2_(*t*_*m*_), thermal stress field of a fractured rock during the insolation period could be expressed as:

21 $$ \sigma_{ij}^{t} = \sum\limits_{m = 1}^{M} {q_{2} \left( {t_{m} } \right)\sigma_{ij}^{ * } } $$

Thermal stress field caused by a triangular a fracture line heat source could be expressed as:

22 $$ \sigma_{ij}^{tl} = \int_{\Gamma } {\sigma_{ij}^{t} } {\text{d}}\Gamma $$

For a semi-infinite rock, boundary conditions can be satisfied by mirror image method except for shear stress on the boundary (*σ*_*xz*_ and *σ*_*yz*_). Thermal stress field produced by superposition of all *k* heat sources (line heat sources and mirror heat sources) could be expressed as:

23 $$ \sigma_{1ij} = \sum\limits_{l = 1}^{k} {\sigma_{ij}^{tl} } $$

The shear stresses on the boundary (*σ*_*xz*_ and *σ*_*yz*_) caused by line heat sources and mirror heat sources are the same. Therefore, solutions of the following boundary value problem should be superimposed on the half space problem:

24 $$ \left\{ {\begin{array}{*{20}l} {f_{x} = \left. { - 2\sigma_{1xz} } \right|_{z = 0} } \hfill \\ {f_{y} = \left. { - 2\sigma_{1yz} } \right|_{z = 0} } \hfill \\ \end{array} } \right. $$

where *f*_*x*_, *f*_*y*_ are sum of the shear stresses on boundary caused by line and mirror heat sources (MPa).

Stress field in half space created by concentrated shear stress *f*_*x*_(*ξ*, *η*, 0), *f*_*y*_(*ξ*, *η*, 0) acting on the point (*ξ*, *η*, 0) of boundary could be calculated by:

25 $$ \sigma_{ij}^{f} = A_{ij} \left( {x - \xi ,y - \eta ,z} \right)f_{x} \left( {\xi ,\eta ,0} \right) + B_{ij} \left( {x - \xi ,y - \eta ,z} \right)f_{y} \left( {\xi ,\eta ,0} \right) $$

where $A_{ij} \left( {x - \xi ,y - \eta ,z} \right)$ and $B_{ij} \left( {x - \xi ,y - \eta ,z} \right)$ are stress influence coefficients.

As a result of integration of the influence coefficients $A_{ij} \left( {x - \xi ,y - \eta ,z} \right)$ and $B_{ij} \left( {x - \xi ,y - \eta ,z} \right)$ with respect to *ξ* and *η*, $\overline{A}_{ij} \left( {x - \xi ,y - \eta ,z} \right),\overline{B}_{ij} \left( {x - \xi ,y - \eta ,z} \right)$ could be obtained by:

26 $$ \begin{aligned} \overline{A}_{xx} & = - \frac{1}{2\pi }\left\{ {\frac{{\left( {1 - 2\mu } \right)R^{\prime}z^{2} - \left( {z + 2\mu R^{\prime}} \right)\left( {x - \xi } \right)^{2} }}{{R^{\prime}\left( {R^{\prime} + z} \right)\left[ {\left( {x - \xi } \right)^{2} + z^{2} } \right]}}\left( {y - \eta } \right) - 2\ln \left( {R^{\prime} + y - \eta } \right)} \right\} \\ \overline{A}_{yy} & = - \frac{1}{2\pi }\left[ {\frac{{z + 2\mu R^{\prime}}}{{R^{\prime}\left( {R^{\prime} + z} \right)}}\left( {y - \eta } \right) - 2\mu \ln \left( {R^{\prime} + y - \eta } \right)} \right] \\ \overline{A}_{zz} & = \frac{{\left( {y - \eta } \right)z^{2} }}{{2\pi R^{\prime}\left[ {\left( {x - \xi } \right)^{2} + z^{2} } \right]}} \\ \overline{A}_{xy} & = - \frac{1}{2\pi }\left[ {\frac{{z + 2\mu R^{\prime}}}{{R^{\prime}\left( {R^{\prime} + z} \right)}}\left( {x - \xi } \right) - 2\ln \left( {R^{\prime} + x - \xi } \right)} \right] \\ \overline{A}_{yz} \left( {\xi ,\eta } \right) & = - \frac{z}{{2\pi R^{\prime}}} \\ \overline{A}_{zx} & = - \frac{1}{2\pi }\left\{ {\arctan \left[ {\frac{{\left( {x - \xi } \right)\left( {y - \eta } \right)}}{{R^{\prime}z}}} \right] - \frac{{\left( {x - \xi } \right)\left( {y - \eta } \right)z}}{{R^{\prime}\left[ {\left( {x - \xi } \right)^{2} + z^{2} } \right]}}} \right\} \\ \overline{B}_{xx} & = - \frac{1}{2\pi }\left[ {\frac{{z + 2\mu R^{\prime } }}{{R^{\prime } \left( {R^{\prime } + z} \right)}}\left( {x - \xi } \right) - 2\mu \ln \left( {R^{\prime } + x - \xi } \right)} \right] \\ \overline{B}_{yy} & = - \frac{1}{2\pi }\left\{ {\frac{{\left( {1 - 2\mu } \right)R^{\prime } z^{2} - \left( {z + 2\mu R^{\prime } } \right)\left( {y - \eta } \right)^{2} }}{{R^{\prime } \left( {R^{\prime } + z} \right)\left[ {\left( {y - \eta } \right)^{2} + z^{2} } \right]}}\left( {x - \xi } \right) - 2\ln \left( {R^{\prime } + x - \xi } \right)} \right\} \\ \overline{B}_{zz} & = \frac{{\left( {x - \xi } \right)z^{2} }}{{2\pi R^{\prime } \left[ {\left( {y - \eta } \right)^{2} + z^{2} } \right]}} \\ \overline{B}_{xy} & = - \frac{1}{2\pi }\left[ {\frac{{z + 2\mu R^{\prime } }}{{R^{\prime } \left( {R^{\prime } + z} \right)}}\left( {y - \eta } \right) - 2\ln \left( {R^{\prime } + y - \eta } \right)} \right] \\ \overline{B}_{yz} & = - \frac{1}{2\pi }\left\{ {\arctan \left[ {\frac{{\left( {x - \xi } \right)\left( {y - \eta } \right)}}{{R^{\prime } z}}} \right] - \frac{{\left( {x - \xi } \right)\left( {y - \eta } \right)z}}{{R^{\prime } \left[ {\left( {y - \eta } \right)^{2} + z^{2} } \right]}}} \right\} \\ \overline{B}_{zx} & = - \frac{z}{{2\pi R^{\prime } }} \\ \end{aligned} $$

where $$R^{\prime } = \sqrt {\left( {x - \xi } \right)^{2} + \left( {y - \eta } \right)^{2} + z^{2} }$$.

The boundary surface can be discretized into *S* rectangular elements. Assuming the shear stress at each element is uniformly distributed, the stress field in half space caused by shear stress on the boundary could be obtained by:

27 $$ \sigma_{2ij} = \left. {\left. {\sum\limits_{s = 1}^{S} {\left[ {\overline{A}_{ij} f_{sx} + \overline{B}_{ij} f_{sy} } \right]} } \right|_{{\xi_{s1} }}^{{\xi_{s2} }} } \right|_{{\eta_{s1} }}^{{\eta_{s2} }} $$

where *s* is the *s*th rectangular element , *f*_*sx*_ and *f*_*sy*_ are values of *f*_*x*_ and *f*_*y*_ at center of the *s*th element, respectively, *ξ*_*s*1_, *ξ*_*s*2_ are region restriction in *x* direction of the *s*th element, *η*_*s*1_, *η*_*s*2_ are region restriction in *y* direction of the *s*th element.

Thermal stress field of a single fracture tip in a high-temperature environment could be obtained by

28 $$ \overline{\overline{\sigma }}_{ij} = \sigma_{1ij} + \sigma_{2ij} $$

In the case of *N* fractures in a rock, Eq. (28) represents the thermal stress field caused by the *n*th fracture. The thermal stress field of a multi-fractured rock could be obtained by

29 $$ \overline{\overline{\sigma }}_{ij}^{N} = \sum\limits_{n = 1}^{N} {\overline{\overline{\sigma }}_{ij} } $$

A brittle fracture of a rock satisfies the theory of maximum tensile stress. Stress intensity factors of a surface fracture could be expressed as:

30 $$ \left\{ {\begin{array}{*{20}l} {K_{{\text{I}}} \, = \, \kappa \sigma_{yy} \sqrt {L\pi } } \hfill \\ {K_{{{\text{II}}}} \, = \, \sigma_{yz} \sqrt {L\pi } } \hfill \\ \end{array} } \right. $$

where *K*_I_ and *K*_II_ are stress intensity factors of a fracture, *κ* is correction coefficient of stress intensity factor of a surface fracture.

Local coordinate system is established using a fracture tip point as origin. Vertical fracture direction is *y*′ direction, fracture extension direction is *z*′direction, and direction perpendicular to *y*′*z*′ plane is *x*′ direction. Coordinates can be converted as:

31 $$ \left\{ {\begin{array}{*{20}l} {x^{\prime } = z - L} \hfill \\ {y^{\prime } = y} \hfill \\ {z^{\prime } = x} \hfill \\ \end{array} } \right. $$

Circumferential tensile stress near a fracture tip could be expressed as:

32 $$ \sigma_{\theta } = \frac{1}{{2\sqrt {2\pi r} }}\cos \frac{\theta }{2}\left[ {K_{{\text{I}}} \left( {1 + \cos \theta } \right) - 3K_{{{\text{II}}}} \sin \theta } \right] $$

where *r*, *θ* are polar diameter and polar angle in coordinate system *x*′*oy*′, *x*′ = *r*cos*θ*, *y*′ = *r*sin*θ*.

In case *σ*_*θ*_ reaches the maximum value, fracture initiation angle *θ*_*c*_ satisfies ${{\partial \sigma_{\theta } } \mathord{\left/ {\vphantom {{\partial \sigma_{\theta } } {\partial \theta }}} \right. \kern-0pt} {\partial \theta }}{ = }0,{{\partial^{2} \sigma_{\theta } } \mathord{\left/ {\vphantom {{\partial^{2} \sigma_{\theta } } {\partial \theta^{2} }}} \right. \kern-0pt} {\partial \theta^{2} }} \le 0$. *θ*_*c*_ and *σ*_*θ*max_ could be obtained as follows:

33 $$ \theta_{c} = 2\arctan \frac{{\left[ {1 - \sqrt {1 + 8\left( {{{K_{{{\text{II}}}} } \mathord{\left/ {\vphantom {{K_{{{\text{II}}}} } {K_{{\text{I}}} }}} \right. \kern-0pt} {K_{{\text{I}}} }}} \right)^{2} } } \right]}}{{4\left( {{{K_{{{\text{II}}}} } \mathord{\left/ {\vphantom {{K_{{{\text{II}}}} } {K_{{\text{I}}} }}} \right. \kern-0pt} {K_{{\text{I}}} }}} \right)}} $$

34 $$ \sigma_{\theta \max } = \frac{1}{{\sqrt {2\pi r_{c} } }}\cos \frac{{\theta_{c} }}{2}\left( {K_{{\text{I}}} \cos^{2} \frac{{\theta_{c} }}{2} - \frac{3}{2}K_{{{\text{II}}}} \sin \theta_{c} } \right) $$

where *r*_*c*_ is critical dimension of maximum circumferential tensile stress (m).

When maximum circumferential tensile stress exceeds tensile strength of a rock, i.e.$\sigma_{\theta \max } \ge \left[ {\sigma_{m} } \right]$, a fracture will expand.

## Influence of a fracture on rock temperature field

**Figure 4:**
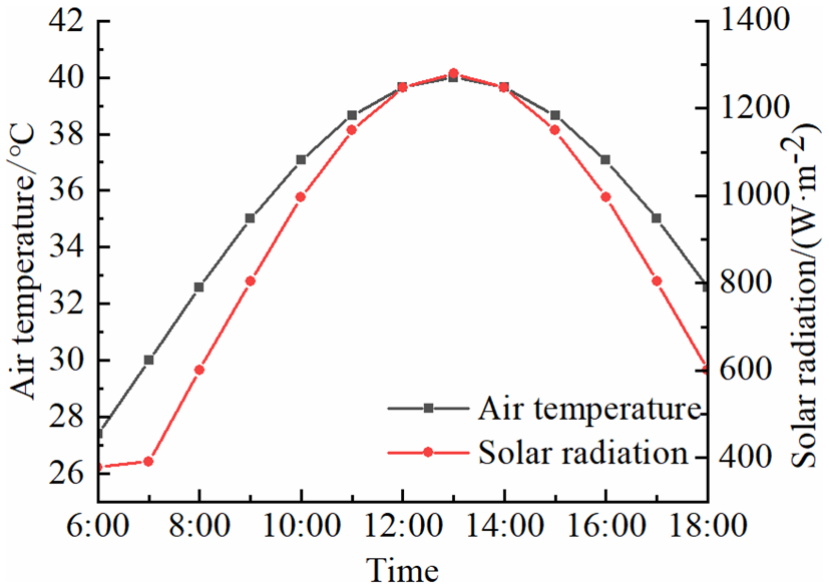
Air temperature and solar radiation^46^.

Fractures are channels through which solar radiation energy is converted into rock thermal energy, affecting rock temperature field. The influence of a fracture on temperature field in a high-temperature environment is studied by using granite in Guangzhou section of the Guangzhou-Shenzhen High-speed Railway as an example. Before sun rises, temperature field of a rock is approximately uniform and this temperature is regarded as the initial rock temperature. Parameters of an environment and granite properties^46^ are shown in Fig. 4 and Table 1. The fracture shape is an isosceles triangle. The length of a fracture is 0.35 m and the length of a fracture opening is 0.14 m.

**Table 1:** Parameters of an environment and granite properties^46^.

Calculate time	Location	Day of year	Temperature/K	Density/(kg m^−3^)
6:00–18:00	113°16′ E 23°06′ N	206	293–313	2.6 × 10^3^
Heat capacity/(J kg^−1^ K^−1^)	Thermal conductivity/(W m^−1^ K^−1^)	Coefficient of thermal expansion/K^−1^	Modulus of elasticity/MPa	Poisson’s ratio
1.1 × 10^3^	2.8	8 × 10^−6^	4 × 10^4^	0.3

### Analysis of temperature field of a fracture tip

**Figure 5:**
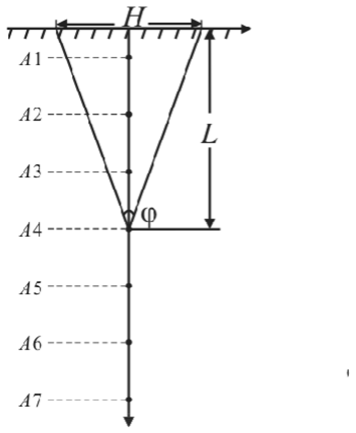
Calculate points of thermal stress.

**Figure 6:**
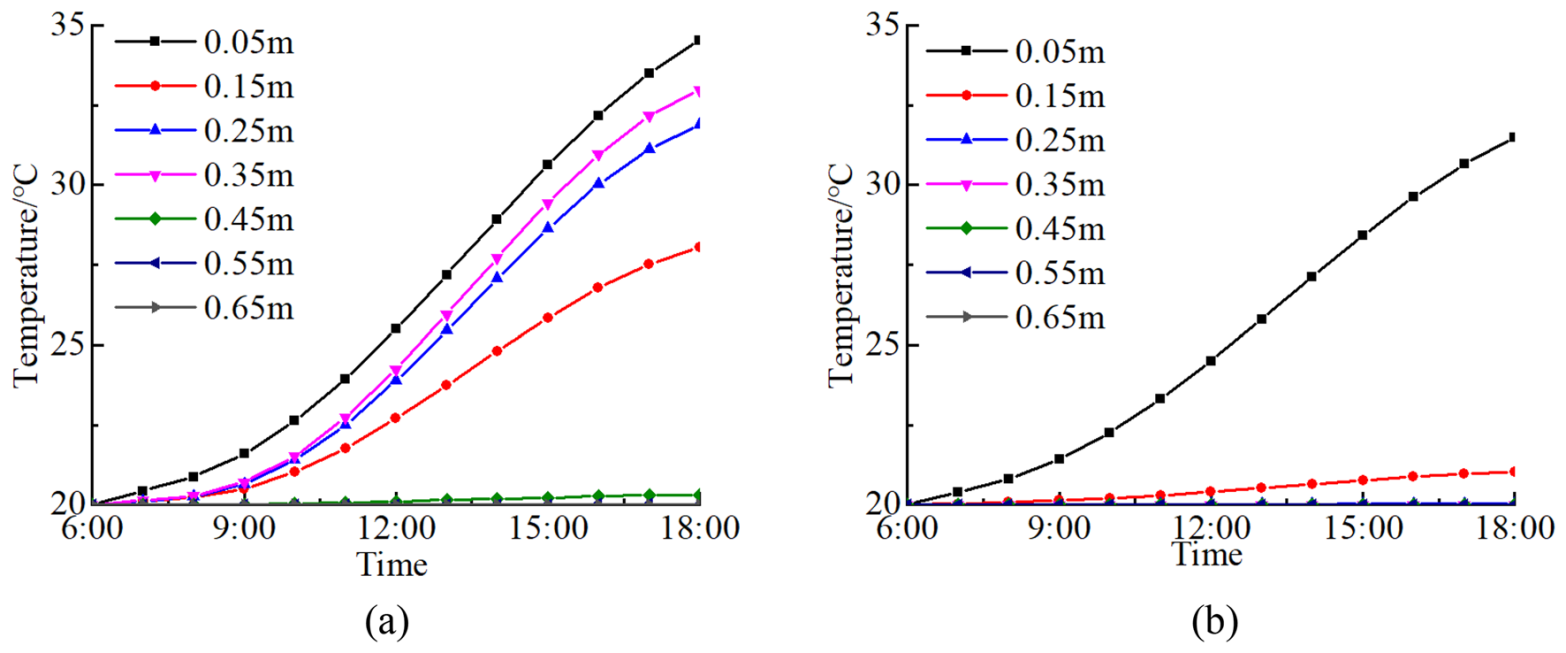
Temperature–time curves of a rock: (**a**) a fractured rock; (**b**) a non-fractured rock.

In solar radiation period, the fractured rock temperatures at depths of 0.05 ~ 0.65 m from the surface (A1 ~ A7 in Fig. 5) are calculated and compared with non-fractured rock temperatures (Fig. 6). During 6:00 ~ 8:00, the difference in temperature between the fractured rock and the non-fractured rock at a given depth is insignificant because of weak radiation. From 9:00 to 18:00, rock temperature increases due to heat effect of solar radiation. The rate of temperature increase is consistent with the changes in solar radiation intensity and reaches its maximum at 13:00. Additionally, the temperature difference between the fractured rock and non-fractured rock increases. Temperature changes of the non-fractured rock are the result of combination of solar radiation, radiation heat transfer and convection heat transfer. When there is a fracture in the rock, convection heat transfer is impossible as the air in a fracture does not flow and the radiation energy cannot escape from the fracture. Therefore, temperature changes of a fractured rock are determined only by the intensity of solar radiation.

The temperature of a fractured rock is higher than that of a non-fractured rock at the same depth, and it varies rapidly and greatly. For a fractured rock under solar radiation, the fracture tip has strong physical and geometric effect, which presents a strong ability to absorb energy and leads to heat and energy concentration. The temperature field of a fracture tip is nonlinear and the temperature of the fracture tip is high. Under the fracture tip, the temperature decreases sharply and eventually approaches the initial rock temperature along *z* axis with depth. In a high-temperature environment, the change in rock temperature field caused by a fracture is primarily confined to the areas around the fracture and has little effect on distant areas.

### Analysis of fracture shape parameters

**Figure 7:**
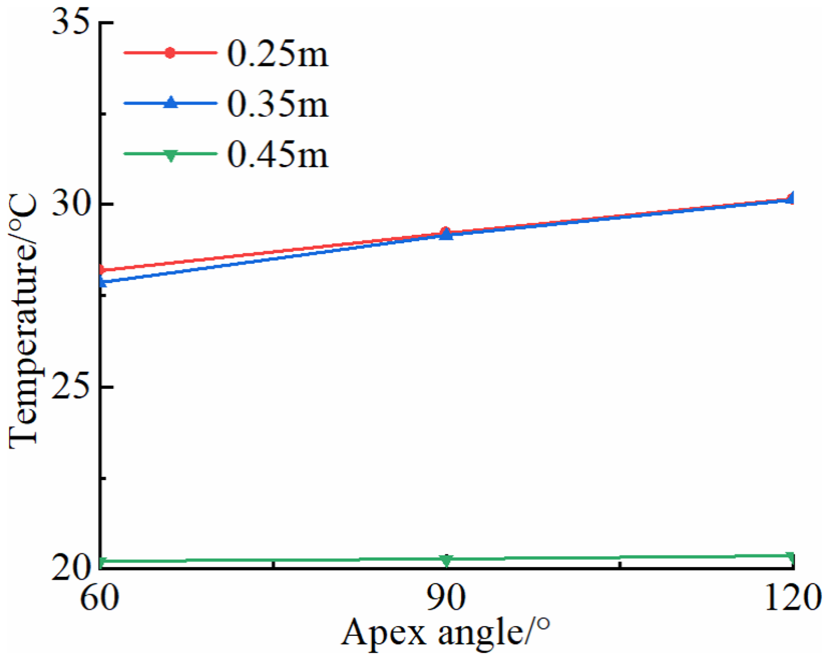
Analysis of temperature change of typical triangular fractured rocks.

In a high-temperature environment, the distribution of rock temperature field is affected by the fracture shape. In order to analyze the influence of shape parameters, temperature fields of typical triangular (acute angle, right angle, obtuse angle) fractured rocks with the same a fracture length of 0.35 m are calculated at 13:00 (Fig. 7). The apex angles of triangular fractures are 60°, 90° and 120°, respectively. The rock temperature increases with the increase of apex angle of a triangular fracture and reaches its maximum when the apex angle is 120°. It is observed that the temperature of the triangular fractured rocks with a constant fracture length vary greatly when the apex angle is large in a high-temperature environment.

## Influence of a fracture on rock thermal stress field

### Analysis of thermal stress field of a fracture tip

**Figure 8:**
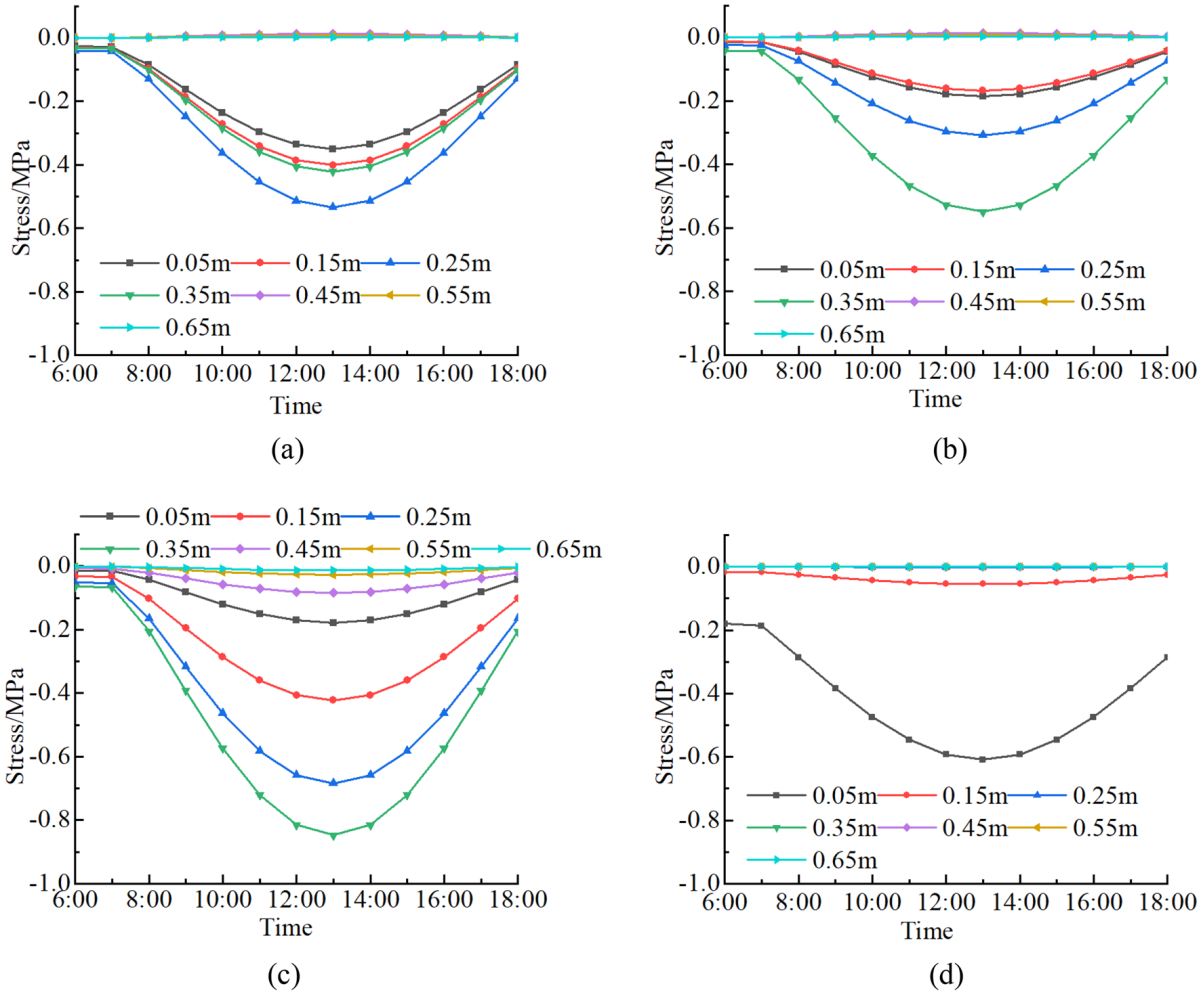
Thermal stress-time curves of a rock: (**a**) *σ*_*x*_ of a fractured rock; (**b**) *σ*_*y*_ of a fractured rock; (**c**) *σ*_*z*_ of a fractured rock; (**d**) *σ*_*x*_ and *σ*_*y*_ of a non-fractured rock.

In order to examine the impact of a fracture on rock thermal stress field in a high-temperature environment, thermal stress of a fractured rock at depths of 0.05 ~ 0.65 m from surface (A1 ~ A7 in Fig. 5) is calculated and compared with that of a non-fractured rock (Fig. 8). In the fractured rock and the non-fractured rock, the daily variation trends of thermal stress are all consistent with that of the solar radiation. When the solar radiation is weak between 6:00 and 8:00, the thermal stress of the fractured rock and the non-fractured rock are basically unchanged. From 9:00 to 13:00, when the solar radiation increases gradually, the thermal stress increases sharply at first and then tends to be gentle. At 13:00, the maximum of thermal stress occurs. From 14:00 to 18:00, when the solar radiation weakens, the thermal stress gradually decreases.

The thermal stress field of a rock is redistributed due to a fracture. Horizontal stresses *σ*_*x*_, *σ*_*y*_ of a non-fractured rock concentrate at the surface layer and decrease rapidly to 0 as depth increases. Vertical stress *σ*_*z*_ of a non-fractured rock is 0. Compared with a non-fractured rock, the stress concentration area in a fractured rock is formed near the fracture tip. The *σ*_*x*_ first increases and then decreases with depth and the maximum value is 0.53 MPa at the depth of 0.25 m. The maximum value of fracture tip(A4) is 0.42 MPa. The *σ*_*y*_ and *σ*_*z*_ increase with depth, and the maximum values are 0.55 and 0.85 MPa at fracture tip, respectively. Under the fracture tip, the thermal stress decreases sharply with depth and finally approaches 0. Fractures provide channels for transfer and transformation of solar radiation energy to the interior of rocks, which is equivalent to adding linear heat sources in rocks. Therefore, fractures change the internal heat conduction law and redistribute the thermal stress field of rocks. Compared with a non-fractured rock, horizontal stress *σ*_*x*_, *σ*_*y*_ of a fractured rock are larger and vertical stress *σ*_*z*_ occurs in a fractured rock. Thus, thermal stress of a fractured rock at the depth of fracture tip increases compared with non-fractured rock in the same depth and thermal stress field near a fracture tip is nonlinear, which is easy to cause cracking and lead to rock instability. The change in rock thermal stress field caused by a fracture in a high-temperature environment is concentrated in areas around the fracture and has little influence on distant areas.

**Figure 9:**
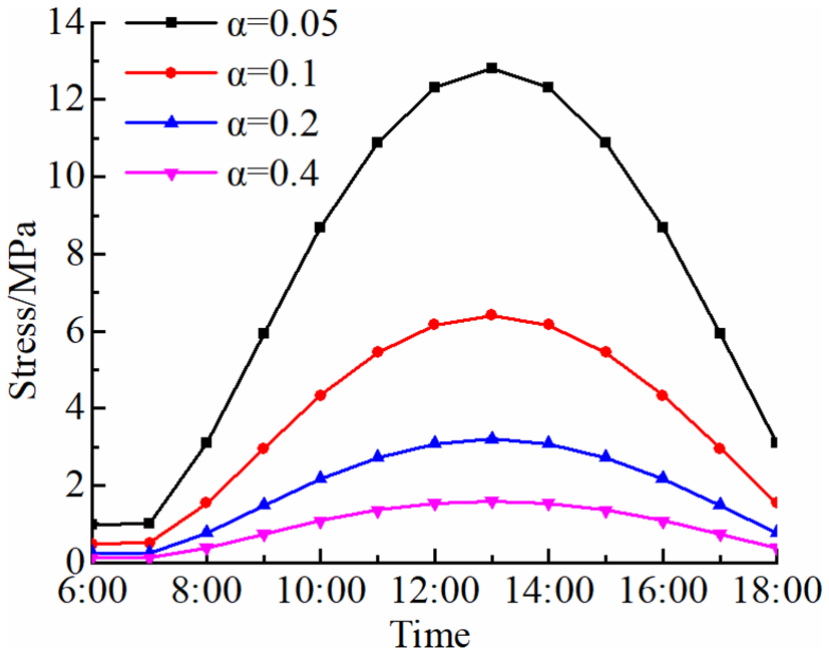
Influence of relative critical size *α* on maximum circle tensile stress *σ*_*θ*max_.

The circumferential tensile stress near a fracture tip is analyzed using relative critical size $\alpha = \sqrt {{{2r_{c} } \mathord{\left/ {\vphantom {{2r_{c} } H}} \right. \kern-0pt} H}}$. The results are equivalent when either critical size *r*_*c*_ or relative critical size *α* approaches 0. The influence of *α* on maximum circumferential tensile stress *σ*_*θ*max_ is demonstrated in Fig. 9. Since the relative critical size *α* is small, the maximum circumferential tensile stress *σ*_*θ*max_ is great. In other words, the change rate and daily amplitude variation of *σ*_*θ*max_ is great near a fracture tip. During the period of insolation, the gradient of *σ*_*θ*max_ increases first and then decreases, reaching its maximum at 13:00.

### Analysis of fracture shape parameters

**Figure 10:**
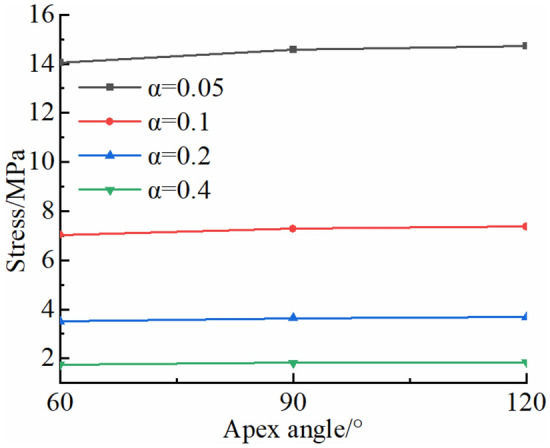
*σ*_*θ*max_ of typical triangular fractured rocks.

In a high-temperature environment, thermal stress of a rock is affected by fracture shape. For analysis of the influence of fracture shape parameters on thermal stress field of a rock, the maximum circumferential tensile stresses *σ*_*θ*max_ near tips of typical triangular (acute angle, right angle and obtuse angle) fractures with the same length of 0.35 m are calculated. The apex angles of triangular fractures are 60°, 90° and 120°, respectively. The changes in *σ*_*θ*max_ of three typical triangular fractured rocks are analyzed at 13:00 when the thermal stress reaches its maximum (Fig. 10). For rocks with triangular fractures of the same length in a high-temperature environment, the *σ*_*θ*max_ increases with the increase in apex angles of triangular fractures, reaching a maximum when the apex angle is 120°. The stress gradient is high as the apex angle of triangle is large. Additionally, *σ*_*θ*max_ does not change greatly as *α* increases.

### Analysis of fracture interaction

Numerous fractures are disorderly distributed in an engineering rock mass. Interaction between fractures is characterized by a superposition of stress fields of fracture tips resulting in fracture connection and rock instability. Considering the effect of fracture number and distribution on thermal stress field, maximum circumferential tensile stress *σ*_*θ*max_ near fracture tips are studied for a rock with double-fracture and triple-fracture in a high-temperature environment.

#### Parallel fractures interaction

**Figure 11:**
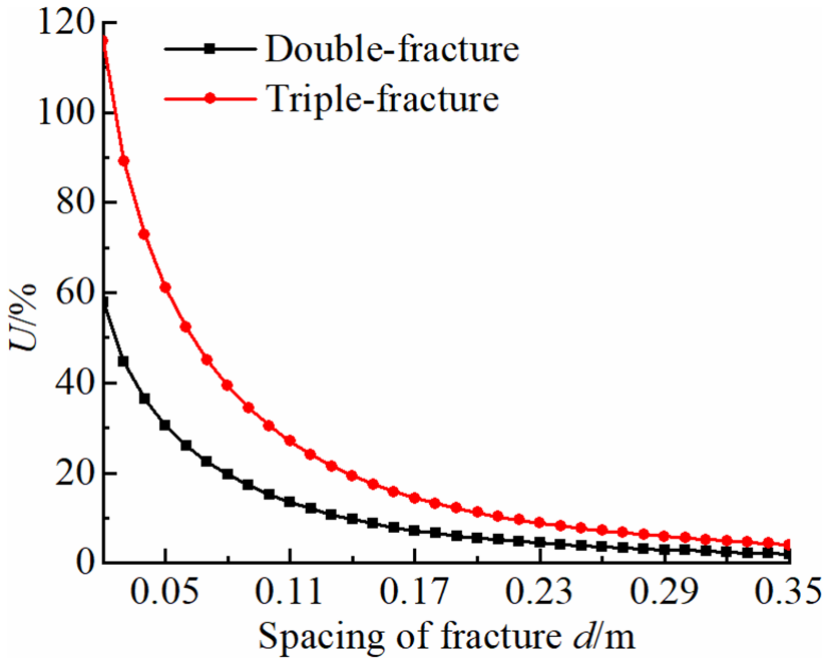
Interaction of stress field of parallel fractures.

The maximum circumferential tensile stress *σ*_*θ*max_ near a tip of parallel fractures is calculated and the change in stress compared to a single fracture is analyzed at 13:00 with a length of fracture of 0.35 m and a length of fracture opening of 0.14 m (Fig. 11). Vertical spacing *d* is 0.02, 0.04, 0.06, 0.08, 0.10, 0.12 and 0.14 m, respectively. *U* is set as percentage change in stress between multi-fracture compared to a single fracture. The thermal stress field of a fracture tip is superposed due to interaction of fractures. Fracture spacing affects the superposition. Maximum circumferential tensile stress *σ*_*θ*max_ is analyzed by setting *V* as a ratio of a vertical fracture spacing *d* to a fracture length *L*. When the distance between parallel fractures is small (*V* ≤ 0.20 of double-fracture, *V* ≤ 0.37 of triple-fracture), the *σ*_*θ*max_ near a fracture tip increases more than 20% and the interaction of fractures produces a significant increase in *σ*_*θ*max_. When there is a certain distance between parallel fractures (0.20 < *V* ≤ 0.60 of double-fracture, 0.37 < *V* ≤ 0.89 of triple-fracture), the fracture interaction is weak. When the distance is long (*V* > 0.60 of double-fracture, *V* > 0.89 of triple-fracture), the *σ*_*θ*max_ near a fracture tip increases less than 5%, which is similar to a single fracture, and the interaction of fractures is not significant.

#### Coplanar fractures interaction

**Figure 12:**
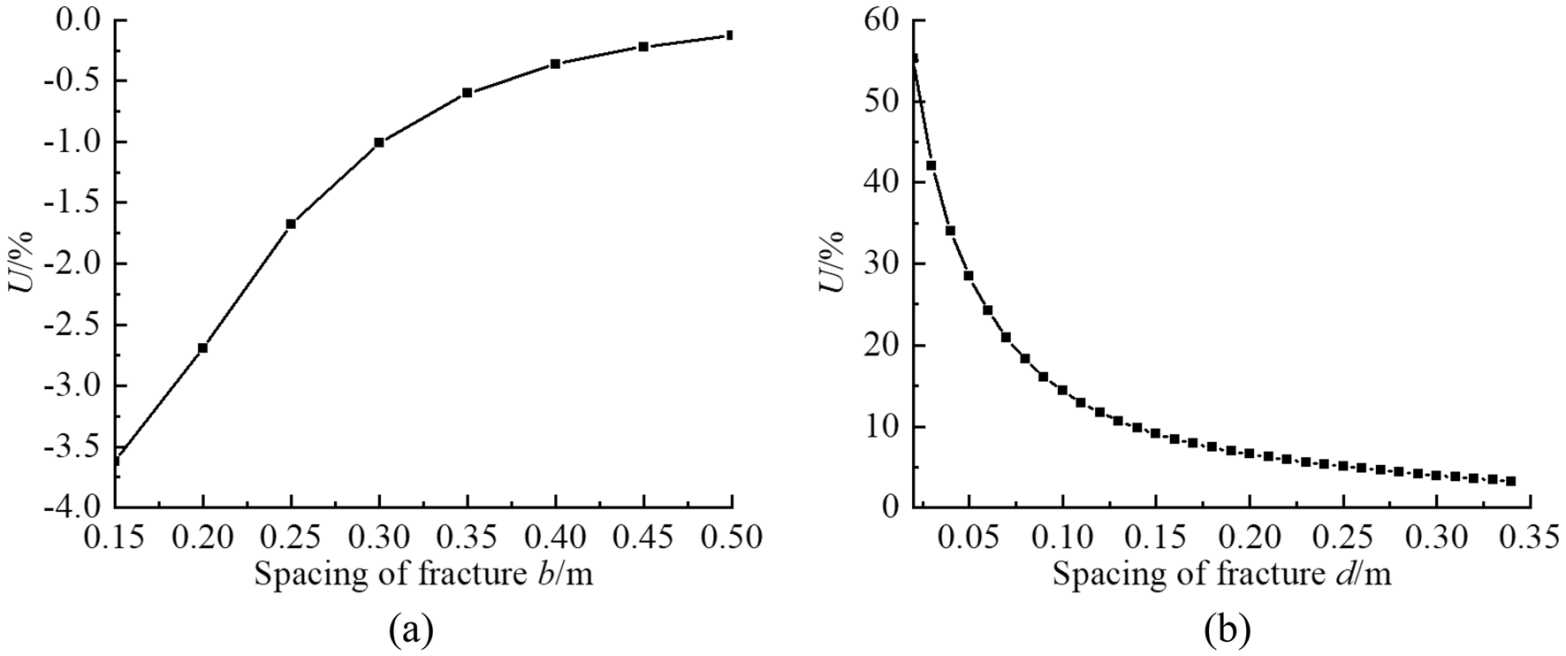
Interaction of stress field of coplanar fractures: (**a**) double-fracture; (**b**) triple-fracture.

The maximum circumferential tensile stress *σ*_*θ*max_ near a tip of coplanar fractures is calculated and the change in stress compared to a single fracture is analyzed at 13:00 with a length of fracture of 0.35 m and a length of fracture opening of 0.14 m (Fig. 12). Horizontal spacing *b* of double-fracture is 0.01, 0.06, 0.11, 0.16, 0.21, 0.26, 0.31, 0.36 m, as well as horizontal spacing *b* of triple-fracture is 0.06 m and vertical spacing *d* is 0.02, 0.04, 0.06, 0.08, 0.10, 0.12 and 0.14 m. The *σ*_*θ*max_ near a tip of coplanar double-fracture does not change significantly as horizontal spacing *b* increases. Fracture interaction is weak and the value of *σ*_*θ*max_ is similar to a single fracture. When the distance between coplanar triple-fracture is small (*V* ≤ 0.20), the *σ*_*θ*max_ near a fracture tip increases more than 20% and the interaction of fractures produces a significant increase in *σ*_*θ*max_. When there is a certain distance between coplanar triple-fractures (0.20 < *V* ≤ 0.71) , the fracture interaction is weak. When the distance is long (*V* > 0.71) , the *σ*_*θ*max_ near a fracture tip increases less than 5%, which is similar to a single fracture, and the interaction of fractures is not significant.

Stress field of a fracture tip is superposed due to fracture interaction. When the spacing between parallel or coplanar fractures is small, the superposition of thermal stress of a fracture tip is significant. As the spacing increases, the value of *σ*_*θ*max_ of multiple fractures tends to be the same as a single fracture.

## Thermal stress field of fractured rocks

The thermal stress field of fractured rocks in a high-temperature environment is analyzed by numerical simulation method. The boundary for heat transfer between a rock and the external environment is complex and variable as the heat exchange process is affected by several natural factors. Furthermore, an engineering rock mass is characterized by nonuniformity and discontinuity due to intensive development of joints and fractures. Therefore, analytical methods are restricted, and numerical simulation methods are effective technical solutions.

### Finite element analysis and related parameters

The finite element model of a fractured rock in the high-temperature environment is 10 m × 10 m × 10 m in size. The triangular fracture develops on upper boundary along *z* direction with a fracture length of 0.35 m and a fracture opening length of 0.14 m. Since the model size is much larger than that of the fracture, the model is approximately satisfied with a semi-infinite body with the fracture. Parameters of an environment and rock properties are shown in Fig. 4 and Table 1. In the finite element analysis, temperature field and thermal stress field satisfy the Fourier heat conduction law and the equilibrium differential equation, respectively. Simulation of temperature field and thermal stress field is performed using solid heat transfer module, surface-to-surface radiation module and solid mechanics module.

**Figure 13:**
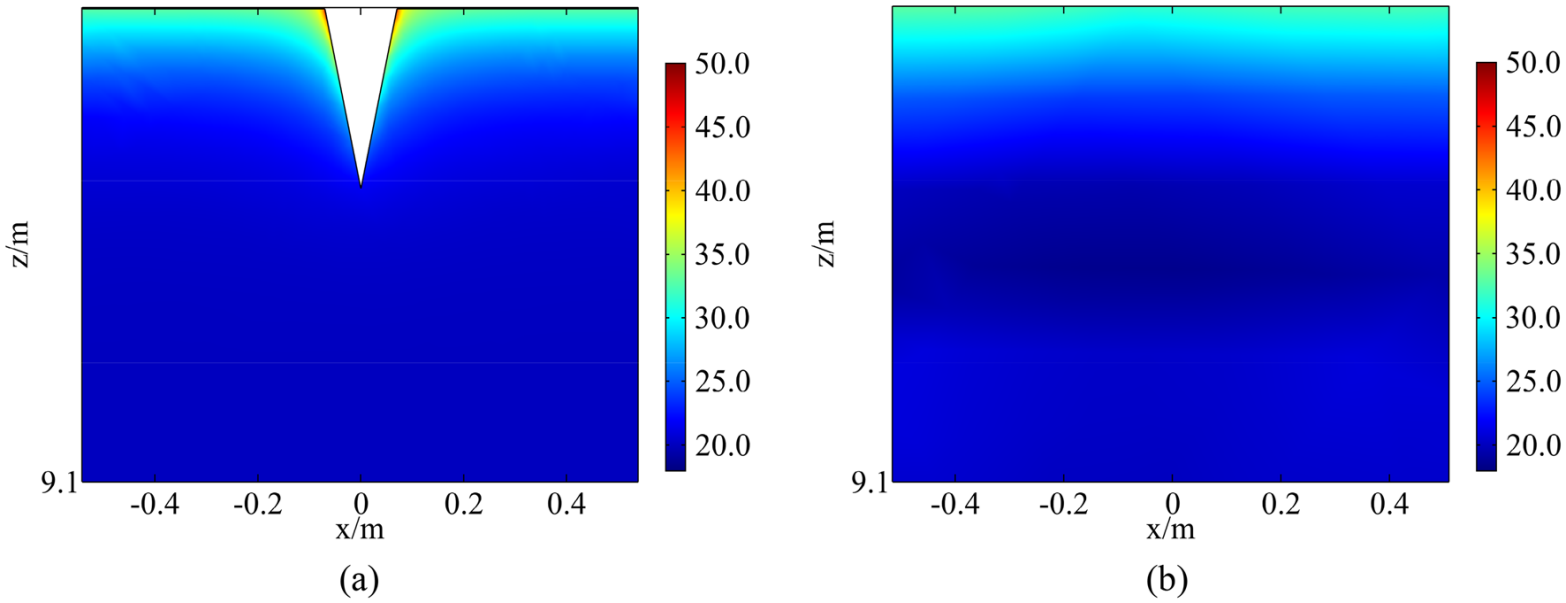
Temperature field of a rock in a high-temperature environment: (**a**) a fractured rock; (**b**) a non-fractured rock (°C).

Temperature field of a fractured rock and a non-fractured rock in a high-temperature environment at 13:00 is obtained, as shown in Fig. 13. It is observed that the temperature field of a fractured rock is nonlinear. Temperature around the fracture increases and isotherms around the fracture tip are concave. Temperature under the fracture tip decreases sharply as depth increases, approaching initial rock temperature. The consistent result of numerical simulation and theoretical analysis indicates that the model is rational. However, as a finite body is used in numerical model to simulate an infinite body, there are numerous fluctuations in temperature field near the boundary leading to a difference between the results of numerical model and theoretical analysis.

### Temperature field of fractured rocks

**Figure 14:**
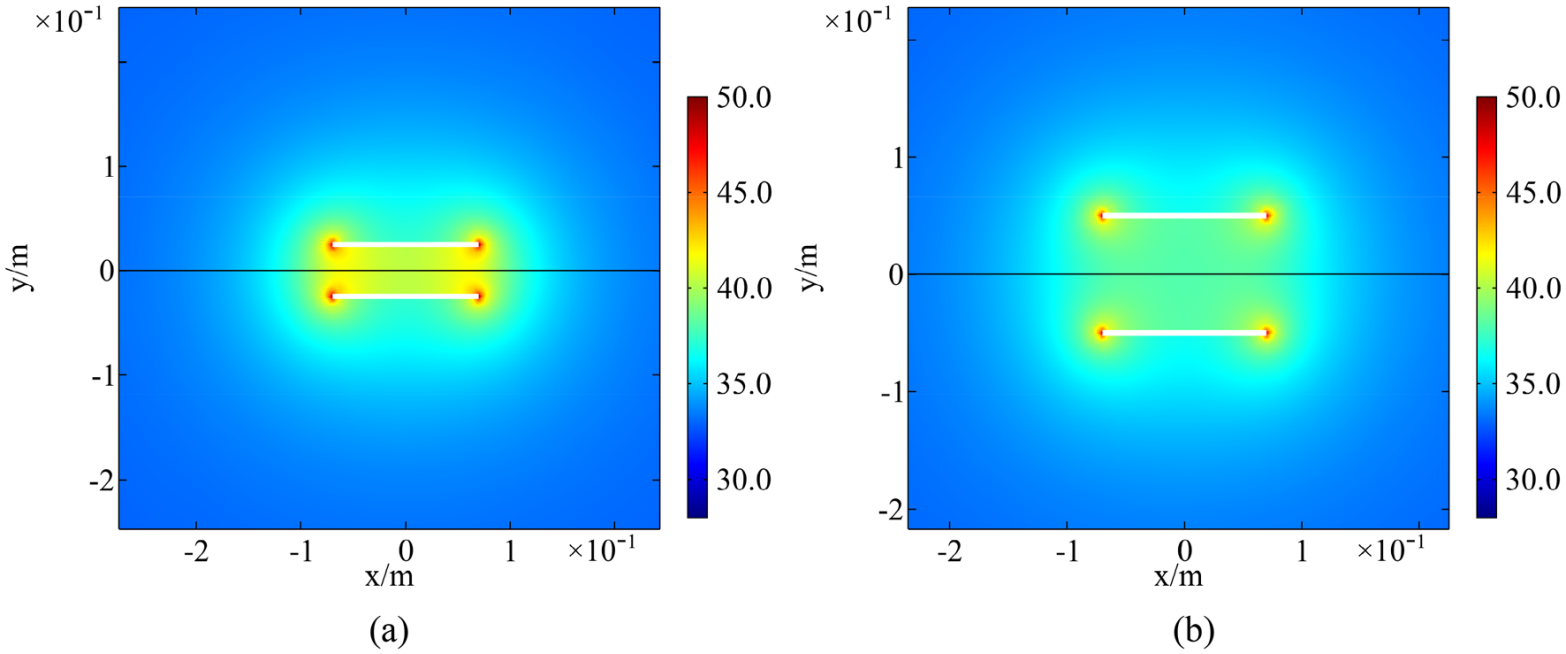
Temperature field of parallel fractures: (**a**) *d* = 0.05 m; (**b**) *d* = 0.10 m (°C).

**Figure 15:**
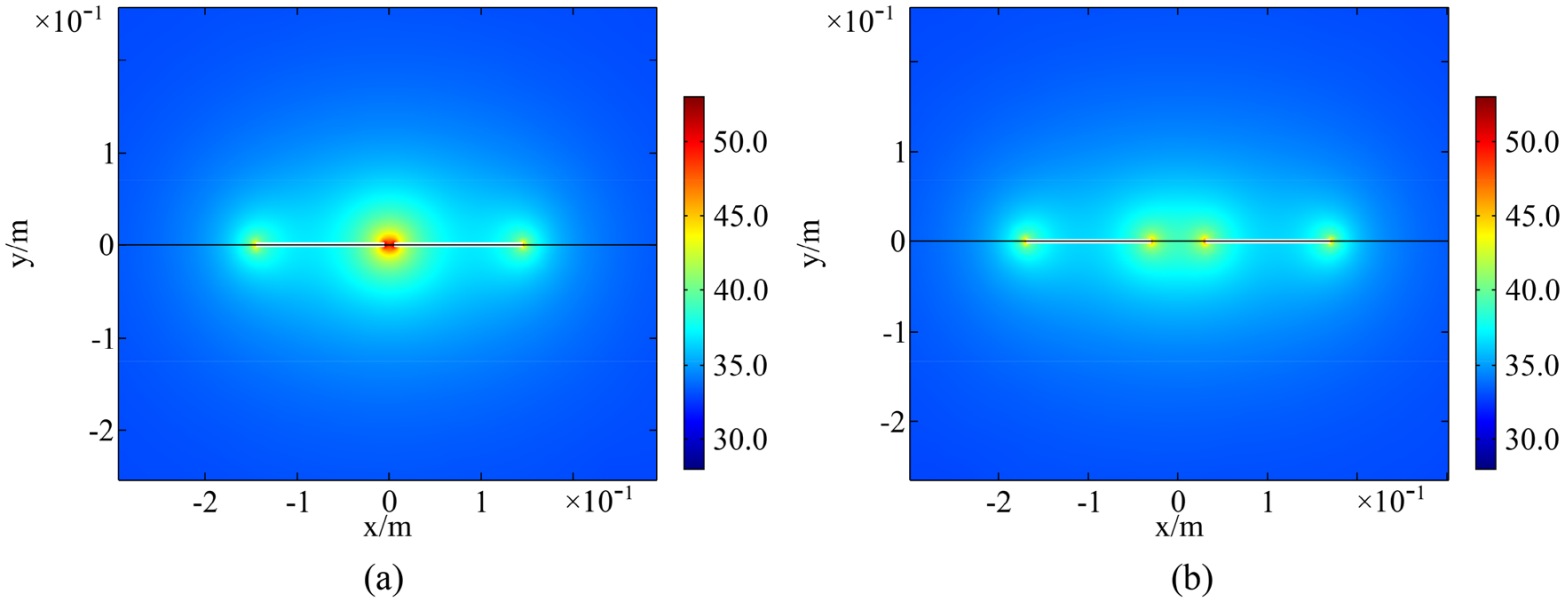
Temperature field of coplanar fractures: (**a**) *b* = 0.01 m; (**b**) *b* = 0.06 m (°C).

Temperature field of a rock with parallel fractures and coplanar fractures at 13:00 in a high-temperature environment is presented in Figs. 14 and 15. The spacing of parallel fractures *d* is 0.05, 0.10 m and the spacing of coplanar fractures *b* is 0.01, 0.06 m, respectively. The upper surface of the numerical model is selected for analysis. The results indicate that the temperature around fractures increases significantly. As distance between parallel fractures and coplanar fractures is small, the interaction of multi-fracture is excellent and the regional temperature field between fractures is significantly enhanced. In the same environment, thermal deterioration is more likely to occur in a multi-fractured rock due to high temperature and large temperature range.

### Thermal stress field of fractured rocks

**Figure 16:**
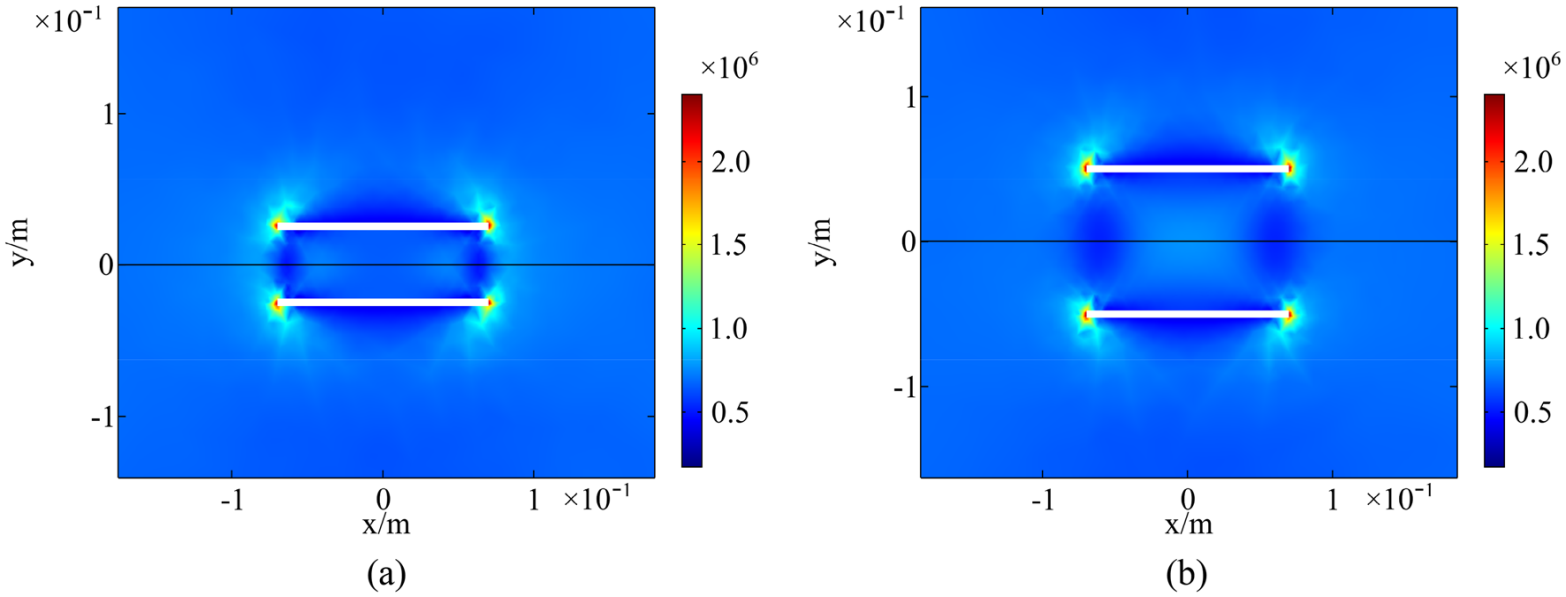
Thermal stress field of parallel fractures: (**a**) *d* = 0.05 m; (**b**) *d* = 0.10 m (Pa).

**Figure 17:**
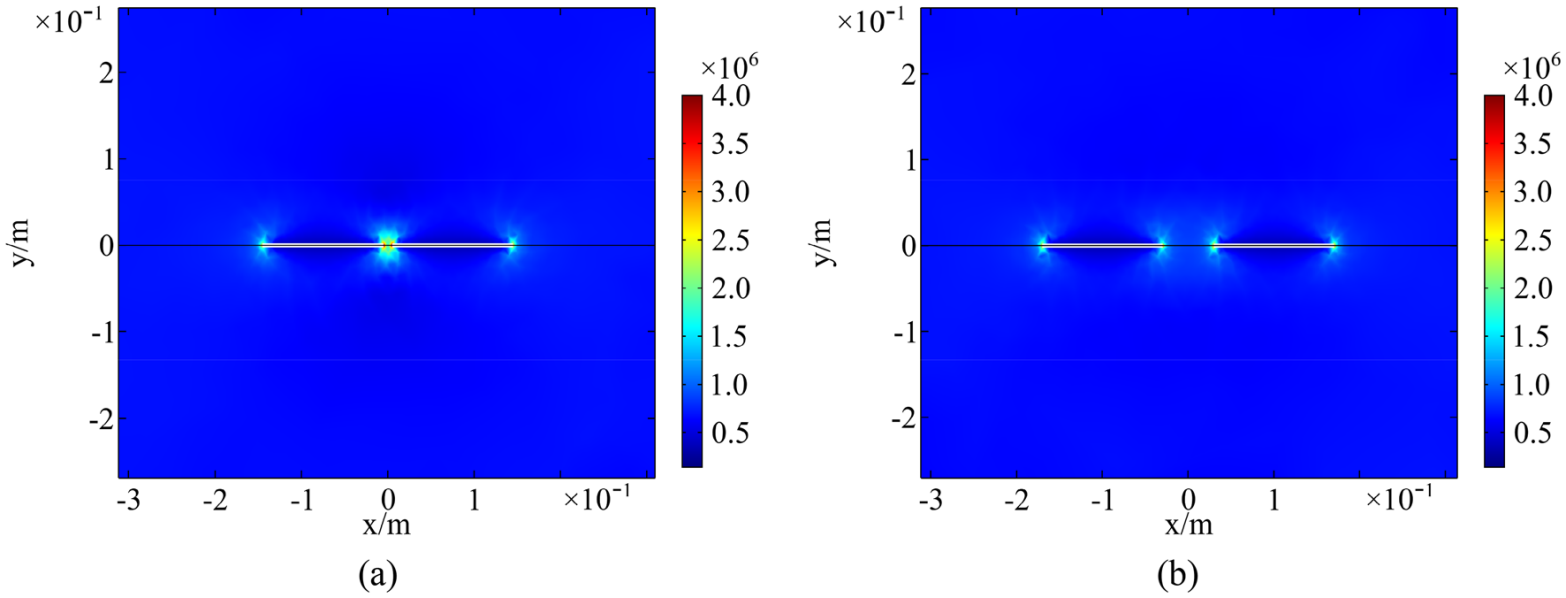
Thermal stress field of coplanar fractures: (**a**) *b* = 0.01 m; (**b**) *b* = 0.06 m (Pa).

Thermal stress field of a rock with parallel fractures and coplanar fractures at 13:00 in a high-temperature environment is shown in Figs. 16 and 17. Spacing of parallel fractures *d* is 0.05, 0.10 m and spacing of coplanar fractures *b* is 0.01, 0.06 m, respectively. An analysis of upper surface of the numerical model suggests a significant increase in thermal stress around the fractures. The interaction of multi-fracture is great and the regional stress field between fractures is significantly enhanced as the distance between parallel fractures and coplanar fractures is small. In the same environment, thermal deterioration is more likely to occur in a multi-fractured rock.

## Case study of rock thermal stress in a tunnel portal

In southern China, a tunnel portal has been exposed to strong solar radiation in a high temperature environment for a long time. Thermal deterioration of fractured rocks occurs as a result of temperature fluctuations and thermal stress concentration. In order to study the changes of fractured rocks thermal stress in a tunnel portal under long-term of solar radiation, the model of this paper is used to analyze the thermal stress of fractured rocks after 5 years and 10 years by numerical calculation.

**Figure 18:**
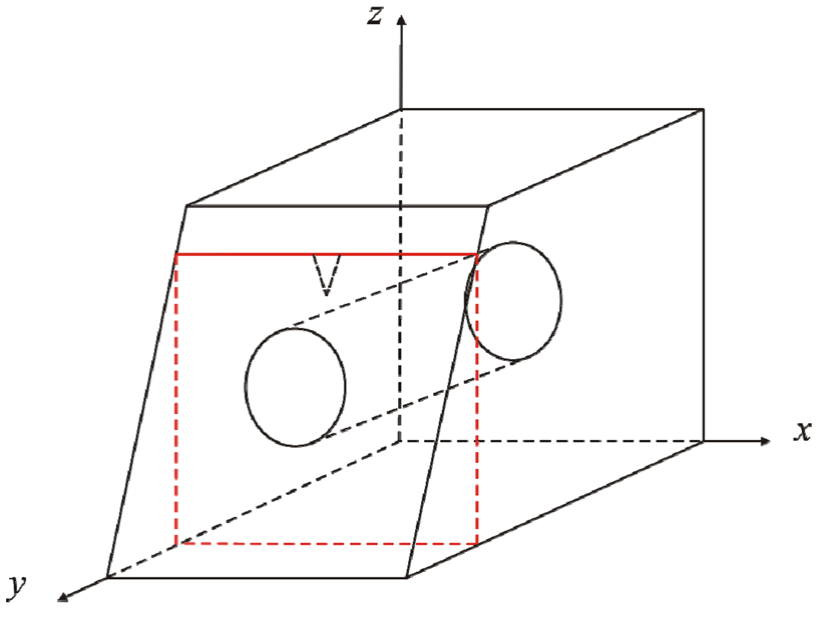
Model of a tunnel portal.

**Figure 19:**
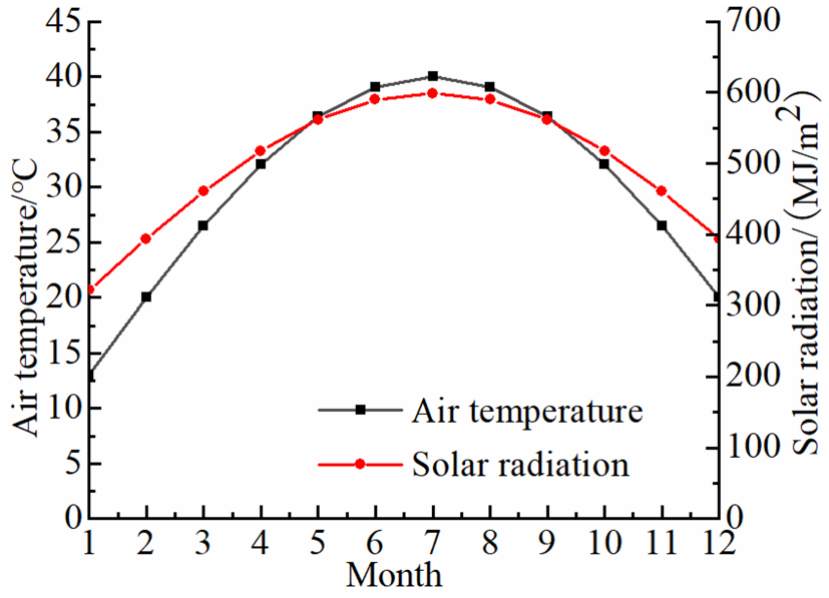
Temperature and solar radiation.

It is assumed that the tunnel has a circular cross-section, and there is a triangular fracture along *z* direction with a fracture length of 0.35 m and a fracture opening length of 0.14 m at 1 m above the tunnel entrance, as shown in Fig. 18. The granite property parameters of the tunnel portal are shown in Table 1, and the annual changes of temperature and solar radiation are shown in Fig. 19. The initial condition of finite element model is the temperature of rocks at the initial moment (initial rock temperature). The boundary condition is the heat exchange between the upper surface of the model and the external environment (Eq. (3)), and the remaining surface is the adiabatic boundary. The boundary of the fracture is the radiation energy absorbed by the fracture (Eq. (8)). The lower boundary of the model is a fixed constraint, and the remaining boundaries can expand and shrink freely.

**Figure 20:**
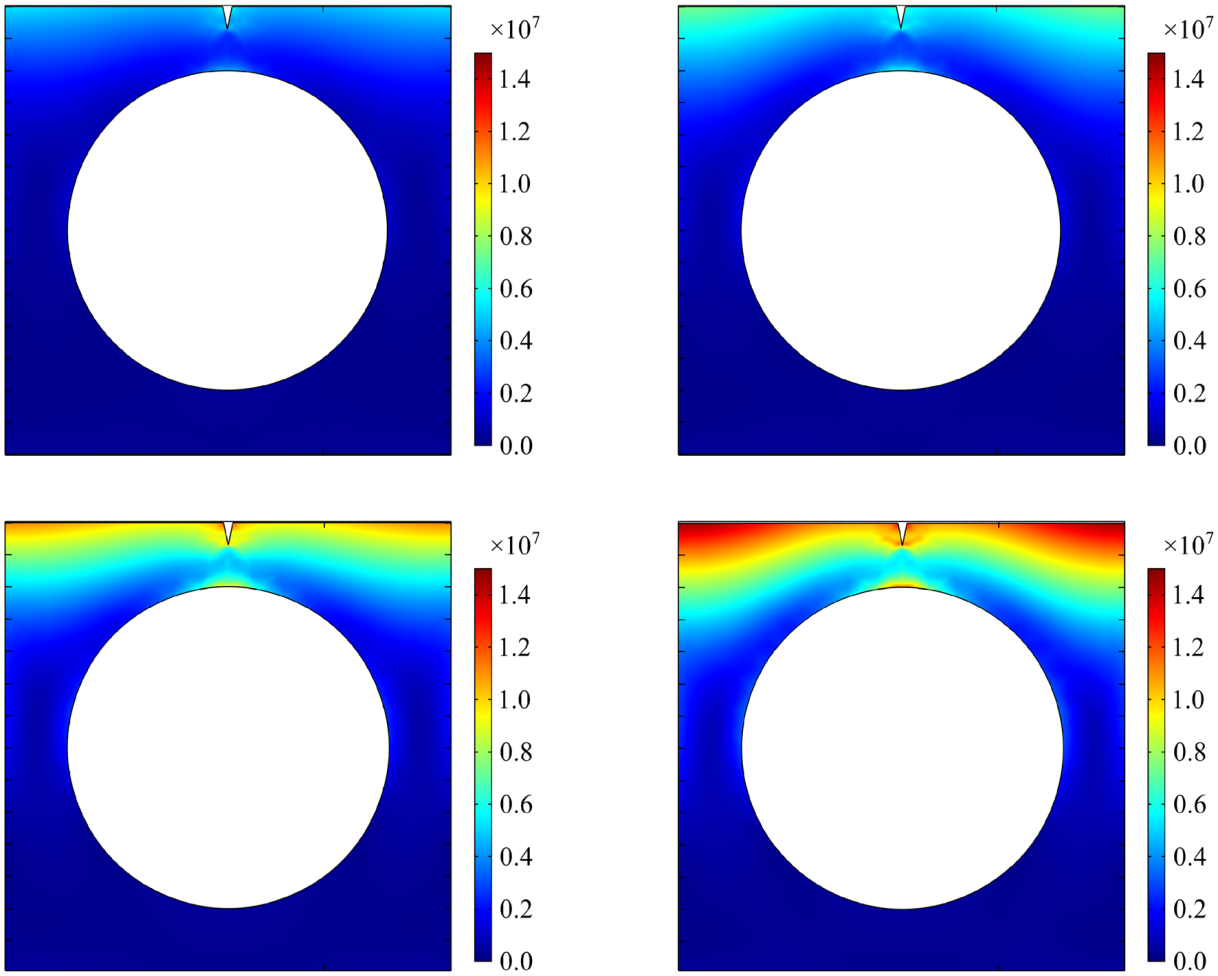
Thermal stress of fractured rock of a tunnel portal (**a**) in February after 5 years; (**b**) in July after 5 years; (**c**) in February after 10 years; (**d**) in July after 10 years (Pa).

The thermal stress of fractured rocks of a tunnel portal after 5 years and 10 years under solar radiation is shown in Fig. 20. The cross section containing the fracture is selected (Fig. 18) for analysis: (1) The thermal stress around the fracture increases significantly, and stress concentration occurs at the fracture tip. Under long-term solar radiation, the thermal stress at the fracture tip increases to 12.3 MPa after 10 years. (2) The thermal stress field around the fracture is nonlinear, and the range of stress redistribution at fracture tip tends to be wide. If a fracture is close to the tunnel portal, it is easy to expand and even penetrate the rock surface, which is a potential hazard source. The results are consistent with the literature^46–50^.

For exposed rocks of tunnel portals in high temperature environments, various measures can be conducted to reduce thermal stress and prevent thermal deterioration, e.g. shielding measures, grouting and anchor stock, which could reduce the direct solar radiation to rocks or enhance rock mechanical properties.

## Conclusions

In this paper, a function expression of triangular heat sources of a fractured rock in a high-temperature environment is established. By the function expression, the nonlinear temperature field and thermal stress field of rock fracture tips are calculated. Additionally, influence of fracture shape parameters and fracture interaction is analyzed. The following conclusions are obtained:

1. During insolation period, the internal temperature of a fractured rock is higher than that of a non-fractured rock. The change in rock temperature field caused by a fracture is concentrated in areas around the fracture. Temperature field near a fracture tip is nonlinear. Temperature of a triangular fractured rock increases as the fracture apex angle increases.
2. During insolation period, the thermal stress field of a rock is nonlinear and the stress of fracture tips increases due to fractures. The *σ*_*θ*max_ of a triangular fractured rock increases as the fracture apex angle increases.
3. As a result of fracture interaction, the thermal stress fields of fracture tips are superposed. When spacing between parallel fractures or coplanar fractures is small, the superposition effect of thermal stress field is evident. In parallel double-fracture, parallel triple-fracture, and coplanar triple-fracture, where the ratio of a vertical spacing to a fracture length is less than 0.20, 0.37 and 0.20, respectively, the *σ*_*θ*max_ near a fracture tip increases more than 20% and the interaction of fractures contributes to a significant increase in *σ*_*θ*max_. As the spacing increases, the value of *σ*_*θ*max_ approaches that of a single fracture. The interaction of coplanar double-fracture is weak and the change in value of *σ*_*θ*max_ is not significant.
4. Temperature field and thermal stress field of a fractured rock are both nonlinear in a high-temperature environment. Temperature and thermal stress around fractures increase significantly. The regional temperature field and thermal stress field between fractures are enhanced by multi-fracture interaction. As a result, thermal deterioration is more likely to occur in a multi-fractured rock in the same environment.
5. The thermal stress of fractured rocks in a tunnel portal under the long-term of solar radiation is analyzed in southern China. After 10 years, the thermal stress of the rock fracture tip increases to 12.3 MPa, and the stress redistribution range at fracture tip tends to be wide, which is easy to lead fracture expansion and penetration, forming a potential hazard source.

## Data Availability

Some or all data that support the findings of this study are available from the corresponding authors upon reasonable request.

## References

[1] Smith, B. J. *Weathering Processes and Forms*. In: Geomorphology of Desert Environments (ed. Abrahams, A.D., Parsons, A.J.) 77 (Springer, 1994). 10.1007/978-94-015-8254-4_3

[2] Hall K (1999). The role of thermal stress fatigue in breakdown of rock in cold regions. Geomorphology.

[3] Caputa ZA (2016). The impact of solar radiation on the temperature of the exposed rocks of the karst canyon (the Kraków-Częstochowa Upland, Poland). Bull. Geogr. Phys. Geogr. Ser..

[4] Smith BJ, Srinivasan S, Gomez-heras M, Basheer PAM, Viles HA (2011). Near-surface temperature cycling of stone and its implications for scales of surface deterioration. Geomorphology.

[5] Hoerle S (2006). Rock temperatures as an indicator of weathering processes affecting rock art. Earth. Surf. Process. Landf..

[6] Gunzburger Y, Merrien-Soukatchoff V (2011). Near-surface temperatures and heat balance of bare outcrops exposed to solar radiation. Earth. Surf. Process. Landf..

[7] Chen B, Ding R, Zheng J, Zhang S (2009). Field test on temperature field and thermal stress for prestressed concrete box-girder bridge. Front. Archit. Civ. Eng. China.

[8] Warke PA, Smith BJ (1998). Effects of direct and indirect heating on the validity of rock weathering simulation studies and durability tests. Geomorphology.

[9] Wang T, Yan L (2022). A heat-flux upper boundary for modeling temperature of soils under an embankment in permafrost region. Sci. Rep..

[10] Che, Y. *Introduction to Rock Mass Engineering Geomechanics* 88 (Science Press, 1983). **(in Chinese)** .

[11] Wang Y, Chen W (2021). Study on thermal stress field of triangle fracture of granite in high temperature environment. Chin. J. Rock Mech. Eng..

[12] Wang, Y. & Chen, W. Nonlinear temperature field of granite fracture tip induced by high natural environmental temperature based on fracture shape function. *Rock Soil Mech.* **43**(Suppl 1), 267–274 (2022). 10.16285/j.rsm.2021.1929**(in Chinese)**

[13] Wedekind W, Gross CJ, Hoffmann A, Siegesmund S (2018). Damage phenomenon and petrophysical properties of sandstones at the Phnom Bakheng Temple (Angkor, Cambodia): first investigations and possible conservation measures. Environ. Earth Sci..

[14] Collins BD, Stock GM (2016). Rockfall triggering by cyclic thermal stressing of exfoliation fractures. Nat. Geosci..

[15] Ding M, Huang T, Zheng H, Yang G (2020). Respective influence of vertical mountain differentiation on debris flow occurrence in the Upper Min River. China. Sci. Rep..

[16] Kompaníková Z, Gomez-Heras M, Michňová J, Durmeková T, Vlčko J (2014). Sandstone alterations triggered by fire-related temperatures. Environ. Earth Sci..

[17] Eppes MC, Mcfadden LD, Wegmann KW, Scuderi LA (2010). Cracks in desert pavement rocks: Further insights into mechanical weathering by directional insolation. Geomorphology.

[18] Wu W, Zhu H, Lin JS, Zhuang X, Ma G (2018). Tunnel stability assessment by 3D DDA-keyblock analysis. Tunn. Undergr. Space Technol..

[19] Liu Y, He Z, Li B, Yang Q (2013). Slope stability analysis based on a multigrid method using a nonlinear 3D finite element model. Front. Struct. Civ. Eng..

[20] Wang S, Zhang Z, Wang C, Ren Y (2019). Multistep rocky slope stability analysis based on unmanned aerial vehicle photogrammetry. Environ. Earth Sci..

[21] Zhang, Z. X., Wang, S. F., Huang, X. & Kwok, C. Y. TBM–Block Interaction during TBM tunneling in rock masses: Block classification and identification. *Int. J. Geomech.* **17**(5), E4016001–E4016001.11 (2017). 10.1061/(ASCE)GM.1943-5622.0000640

[22] Wang M, Cao P (2017). Experimental study of crack growth in rock-like materials containing multiple parallel pre-existing flaws under biaxial compression. Geotech. Geol. Eng..

[23] Sun B, Liu S, Zeng S, Wang S, Wang S (2021). Dynamic characteristics and fractal representations of crack propagation of rock with different fissures under multiple impact loadings. Sci. Rep..

[24] Liu, X., Liu, Q., Liu, B., Zhu, Y. & Zhang, P. Failure behavior for rocklike material with cross crack under biaxial compression. *J. Mater. Civ. Eng.* **31**(2), 06018025.1–06018025.8 (2019). 10.1061/(ASCE)MT.1943-5533.0002540.

[25] Wu Z, Li M, Weng L (2020). Thermal–stress–aperture coupled model for analyzing the thermal failure of fractured rock mass. Int. J. Geomech..

[26] Zhai Y, Ma G, Zhao J, Hu C (2008). Dynamic failure analysis on granite under uniaxial impact compressive load. Front. Archit. Civ. Eng. China.

[27] Park J, Park HD (2017). The effect of frost weathering at the dinosaur tracksite in Seoyu-ri, Hwasun. Korea. Bull. Eng. Geol. Environ..

[28] Lin H (2020). Analytical and numerical analysis for frost heaving stress distribution within rock joints under freezing and thawing cycles. Environ. Earth Sci..

[29] Kang Y (2020). Frost deformation and a Quasi–Elastic–Plastic–Creep constitutive model for isotropic freezing rock. Int. J. Geomech..

[30] Li H, Sun S, Wang L, Liu J, Zhang Z (2022). Damage law and mechanism of coal-rock joint structure induced by liquid nitrogen at low temperature. Sci. Rep..

[31] Vinciguerra, S. C., Trovato, C., Meredith, P. G. & Benson, P. M. Relating seismic velocities, thermal cracking and permeability in Mt. Etna and Iceland basalts. *Int. J. Rock Mech. Min. Sci.* **42**(7–8), 900–910 (2005). 10.1016/j.ijrmms.2005.05.022

[32] Zhu Z (2021). Experimental investigation on mechanical behaviors of Nanan granite after thermal treatment under conventional triaxial compression. Environ. Earth Sci..

[33] Liu S, Huang Z (2021). Analysis of strength property and pore characteristics of Taihang limestone using X-ray computed tomography at high temperatures. Sci. Rep..

[34] Peng J, Rong G, Tang ZC, Sha S (2019). Microscopic characterization of microcrack development in marble after cyclic treatment with high temperature. Bull. Eng. Geol. Environ..

[35] Liu J, Zhang Z, Leung AK (2022). Mesoscopic and macroscopic investigation of a dolomitic marble subjected to thermal damage. Sci. Rep..

[36] Wang Z, He A, Shi G, Mei G (2018). Temperature effect on AE energy characteristics and damage mechanical behaviors of granite. Int. J. Geomech..

[37] Nariman NA (2018). Thermal fluid-structure interaction and coupled thermal-stress analysis in a cable stayed bridge exposed to fire. Front. Struct. Civ. Eng..

[38] Alneasan, M. & Alzo’ubi, A. K. Extensive experimental investigation on the effect of thermal treatment and lateral pressure on the shear behavior of intact mudstone. *Sci*. *Rep*. **13**(1), 6820 (2023).‏ 10.1038/s41598-023-33841-5. 10.1038/s41598-023-33841-5 PMC10133224 37100929

[39] Xu L, Gong F, Liu Z (2022). Experiments on rockburst proneness of pre-heated granite at different temperatures: Insights from energy storage, dissipation and surplus. J. Rock Mech. Geotech. Eng..

[40] Zhai T, Zhu J, Zhou C, Yang T (2022). Experimental investigation of the effect of thermal treatment on shear characteristics of healed rock joints. Int. J. Rock Mech. Min. Sci..

[41] Alneasan, M. & Alzo’ubi, A. K. Temperature effect on the fracture behavior of granite under three loading modes (I, I/II, and II). *Rock Mech*. *Rock Eng*. **56**(3), 2197–2211 (2023). 10.1007/s00603-022-03149-3

[42] Yang, S. & Tao, W. *Heat transfer*. 134, 379–383 (Higher Education Press, 2006). (in Chinese)

[43] Yan Z (1984). Analysis of the temperature field in layered pavement system. J. Tongji Univ..

[44] Carslaw, H. S. & Jaeger, J. C. *Conduction of Heat in Solids*. 375–376 (Clarendon Press, 1959).

[45] Takeiwuchi, Y. *Thermal Stress* (Translated by Guo, T., Li, A.) 248–253 (Science Press, 1977). **(in Chinese)**.

[46] Li, M. Thermal-Moisture Stress Analysis of Tunnel Portal Rock in High Temperature and Humidity. Dissertation for the Doctoral Degree, Beijing Jiaotong University (2016) **(in Chinese)**.

[47] Chen W (2016). Analysis of railway tunnel portal section thermal-humidity failure in high temperature and high humidity. J. Rail Eng. Soc..

[48] Li, M. & Chen, W. Analysis thermal stress of a single crack in any direction under dry environment. *Electro. J. Geotech. Eng.* **465**(18), 465:5397–7 (2013)

[49] Li W, Chen W (2023). Mechanisms of coupled mode I–II fracture initiation in rocks subjected to heat flow-induced fracture gas-steam pressure and heat flow stress. Theor. Appl. Fract. Mech..

[50] Li W, Chen W (2022). Linear crack initiation analysis on rock surface under the combined action of sub-elevated temperature stress and fracture air–vapor pressure. Theor. Appl. Fract. Mech..

